# Prevalence of impaired fasting glucose and associated risk factors among Malaysian adult population: Findings from the National Health and Morbidity Survey (NHMS) 2019

**DOI:** 10.1371/journal.pone.0320993

**Published:** 2025-04-16

**Authors:** Murnizar Mokhtar, Nor Azian Mohd Zaki, Nurul Huda Ibrahim

**Affiliations:** Centre for Nutrition Epidemiology Research, Institute for Public Health, National Institute of Health, Ministry of Health of Malaysia, Shah Alam, Malaysia; The Chinese University of Hong Kong, HONG KONG

## Abstract

Impaired fasting glucose (IFG) is a condition when a person’s blood glucose level is above the normal range, but below the diagnostic cut-off for a formal diagnosis of diabetes mellitus. The objective of this study was to determine the prevalence of IFG among adults aged 18 years and older in Malaysia and its’ associated factors. Data were obtained as part of the National Health and Morbidity Survey (NHMS) 2019 [1]. It was a community-based, cross-sectional study that was conducted among a targeted population in both urban and rural areas in all 13 states and 3 federal territories in Malaysia, using a two stage stratified random sampling method. Adults aged 18 years and older with fasting capillary blood glucose (FBG) readings of ≤ 6.9 mmol/L were selected, except those known to have diabetes. IFG was defined according to World Health Organization (WHO) criteria as FBG between 6.1 and 6.9 mmol/L. The data were analyzed using SPSS Version 28.0. A total of 6183 respondents - 2842 men and 3341 women participated in this study. The overall prevalence of IFG was 22.6% (95% CI: 20.4, 24.9). Respondents aged 60 years and older had the highest percentage of IFG at about 30.2% (95% CI: 26.4, 34.4), followed by respondents with hypercholesterolemia at 29.2% (95% CI: 25.2, 33.6) and respondents with hypertension at 27.7% (95% CI: 24.3, 31.4). Multivariate analysis revealed that age of 60 years and above (aOR 1.51, 95% CI: 1.01, 2.06) and marriage (aOR 1.46, 95% CI: 1.16, 1.84) were significantly associated with IFG. The prevalence of IFG among Malaysian adults according to data obtained in 2019 was 22.6%, with an age of 60 and above and married being the associated factors. Policies need to be tailored for more vigorous screening among this group for prompt diagnosis and treatment to prevent complications.

## Introduction

Diabetes mellitus is one of the non-communicable diseases that is known to be the main cause of death in the world. In a worldwide study, adults under the age of 20 in rural Koladiba, Ethiopia participated in a community-based, cross-sectional survey from February to April 2015, which revealed a 12% prevalence [1]. Using fasting and capillary blood glucose measurements taken two hours after a glucose load, a cross-sectional study was conducted in India in 2010 to assess the prevalence of prediabetes among persons aged 20 and older. IFG and/or IGT were associated with prediabetes in 8.3% of people in Tamil Nadu, 12.8% of people in Maharashtra, 8.1% of people in Jharkhand, and 14.6% of people in Chandigarh [2].

Multivariate analysis revealed that age of 60 years and above (aOR 1.51, 95% CI: 1.01, 2.06) and marriage (aOR 1.46, 95% CI:1.16, 1.84) were significantly associated with IFG. The prevalence of IFG among Malaysian adults according to data obtained in 2019 was 22.6% with an age of 60 and above and married being the associated factors. Policies need to be tailored for more vigorous screening among this group for prompt treatment, diagnosis, and treatment to prevent complications. To improve the screening program, considering looking into a potential reason that contributes to an associated factor is recommended. The National Health and Morbidity Survey (NHMS) 2011 and 2015 reported an increasing trend in diabetes prevalence from 11.2% in 2011 to 13.4% in 2015 [3]. According to NNHMS 2019, the prevalence of raised blood glucose among people with unknown diabetes was 8.9%, resulting in the prevalence of overall raised blood glucose in Malaysia in 2019 to be 18.3%. The respondent, who was not known to have diabetes and had a fasting capillary blood glucose (FBG) of 7.0 mmol/L or more (or non-fasting blood glucose of more than 11.1 mmol/L) was considered to have raised blood sugar. Over 80% of those with raised blood glucose but not known to have diabetes were aged 60 years and below.

Impaired fasting glucose occurs when a person’s blood glucose levels are raised but not high enough to be classified as diabetes mellitus. It is defined as an FBG of 5.6–6.9 mmol/L but the exact range varies depending on the guidelines. Studies are currently being carried out to determine how IFG affects mortality and cardiovascular complications. A study was conducted in Korea by analyzing data from a retrospective cohort of the Korean National Health Insurance Service, involving 260,487 Korean adults aged over 40 years without diabetes mellitus or cardiovascular disease at baseline. This study revealed that increasing fasting glucose in the non-diabetic population is associated with risks of Myocardial Infarction (MI), stroke, and all-cause mortality [4]. Another study conducted in 2002 at the French Institute for Health Protection (I.R.S.A.) with 61,724 male and female participants (mean age 40) indicated additional investigation and cardiovascular risk factor prevention for those individuals with fasting blood glucose levels of more than 6.1 mmol/L [5].

In Malaysia, a nationwide cross-sectional study was done in 2018, obtaining data from the National Health and Morbidity Survey (NHMS) 2015, which used fasting capillary blood glucose as a parameter. Using FBG of ≥ 6.1 mmol/L as criteria, this study showed a prevalence of 9.2% of undiagnosed T2DM in Malaysia, with obesity, age, ethnicity, educational level, and hypertensive status as associated factors [6]. A systematic review and meta-analysis were conducted in 2021 to investigate the pooled prevalence of prediabetes and type-2 diabetes in the general population of this country. This study reported an 11.6% prevalence of prediabetes [7]. A cross-sectional study was conducted, including 4982 participants aged 35–70 years old residing in rural and urban areas in Malaysia, as a sub-study under the Prospective Urban Rural Epidemiology (PURE) study, aimed to determine the prevalence of prediabetes and DM and their associated factors among Malaysian adults. This study revealed a prevalence of 10.8% prediabetes with ≥ 50 years old, male, Malay, physically inactive, known hypertension, and family history of DM as associated factors. Prediabetes was defined as finger prick FBS of 5.6 to < 7.0 mmol/L [8]. Another cross-sectional study conducted in 2017 among fishing communities in the Southwest District of Penang, Malaysia, using finger prick tests, reported a prevalence of 10.12% and 19.6% for prediabetes and diabetes, with older age, a bigger waist circumference, and a self-perceived poor routine diet as associated factors with prediabetes [9]. In another study, a combination of FBG and the Oral Glucose Tolerance Test (OGTT) was done among 3879 Malaysian adults to determine the prevalence of prediabetes and diabetes among rural and urban Malaysians. Overall, 22.1% of respondents had prediabetes, and isolated impaired fasting glucose (IFG) and impaired glucose tolerance (IGT) were reported as 3.4 and 16.1%, respectively [10].

The purpose of this study was to determine the prevalence of IFG among adults aged 18 years and older in Malaysia in 2019 and its’ associated factors.

## Materials and methods

The data utilized in this study were obtained from the National Health and Morbidity Survey (NHMS) 2019, a community-based, cross-sectional study. Additional details can be obtained from the National Health and Morbidity Survey (NHMS) 2019: Volume I on Non-Communicable Diseases (NCDs), encompassing an extensive examination of risk factors and various health issues [3]. The survey targeted a diverse sample of adults aged 18 years and above residing in both urban and rural regions across all 13 states and 3 federal territories of Malaysia. This broad geographical coverage ensured representation from various demographic and socioeconomic backgrounds, enhancing the generalizability of the findings. A two-stage stratified cluster sampling study design was utilized in the NHMS 2019 in order to minimize selection bias and capture population diversity. Specifically, 475 Enumeration Blocks (EBs) were selected across Malaysia’s 13 states and 3 federal territories, forming the primary sampling units. From each EB, 12 Living Quarters (LQs) were randomly chosen, resulting in a total of 5700 LQs included in the survey. The response rate of 87.2%, with 14,965 individuals consenting to participate, suggests a high level of engagement and cooperation from the study participants, further enhancing the reliability and validity of the data collected [3].

Data collection for this survey was carried out from July 14th to October 2nd, 2019, using a combination of structured and validated questionnaires alongside clinical assessments. Structured and validated questionnaires were employed to gather demographic, lifestyle, and medical history information from participants, ensuring the accuracy and reliability of the data collected and minimizing measurement errors and biases. Anthropometric assessments were carried out using the Tanita Personal Scale HD 319 for weight and the SECA Stadiometer 213 for height. To ensure precision, each measurement was taken twice, adhering to best practices in measurement protocol. Body Mass Index (BMI) calculations were based on guidelines provided by the World Health Organization (WHO), ensuring consistency and comparability with international standards [11,12]. Clinical and biochemistry assessments involved the use of specialized equipment for blood pressure measurement, fasting blood glucose, cholesterol, and hemoglobin levels [13]. The Omron Japan Model HEM-907 was utilized for blood pressure measurement, while the CardioChek® PA Analyzer and HemoCue® Machine Hb 201 + were employed for assessing fasting blood glucose, cholesterol, and hemoglobin levels, respectively [14,15]. Before the commencement of the study, all anthropometry and clinical measurement equipment underwent rigorous testing and calibration to ensure consistent and precise measurements. Face-to-face interviews, self-administered questionnaires, and clinical assessments were conducted by trained research assistants and nurses to minimize inter-rater variability and ensure standardized data collection procedures across all participants.

Data collection for this manuscript was focused on respondents with a fasting blood glucose level of ≤ 6.9 mmol/L, excluding individuals with known diabetes, to ensure that the study population accurately represented individuals with and at risk for impaired fasting glucose (IFG). The definition of IFG utilized in this study adhered to the classification provided by the World Health Organization (WHO), which delineates IFG as fasting blood glucose levels ranging between 6.1 and 6.9 mmol/L [16]. By employing internationally recognized criteria for defining IFG, the study ensured comparability and consistency with existing literature and clinical practice guidelines. To minimize misclassification and ensure that people with diabetes were excluded from the definition, the term “known diabetes” status was established based on self-reported data or confirmation by a medical practitioner, such as a doctor or assistant medical officer.

### Ethical approval

Data was obtained as part of the National Health and Morbidity Survey (NHMS) 2019. This national survey was registered in the National Medical Research Registry with registration number NMRR-18-3085-44207 and received ethical approval from the Medical Research and Ethics Committee of the Ministry of Health Malaysia. Written consent was obtained from respondents before the survey.

### Statistical analysis

Data were analyzed using SPSS Version 28.0. Socio-demographic characteristics were determined using descriptive analysis, with categorical variables such as gender, age group, and ethnicity presented as frequencies and percentages. Complex sampling design method was used to estimate prevalence, as data were collected from two-stage stratified cluster sampling in all states in Malaysia. We utilized Multiple Logistic Regression analysis to elucidate the impact of various categorical variables within the Malaysian population on the occurrence of impaired fasting glucose. This method enabled us to identify and quantify the associations between these factors and impaired fasting glucose, providing valuable insights into the underlying determinants of this condition within the Malaysian context. To evaluate significant differences between categorical variables, we used Pearson’s chi-square test in addition to Multiple Logistic Regression analysis. As the associated factors vary by nation, this statistical method allowed us to carefully analyze the relationships between categorical characteristics and impaired fasting glucose levels, complementing the findings obtained using logistic regression. Through a thorough analysis of categorical variable distribution, we were able to learn more about how different clinical and demographic factors interact and affect the prevalence of impaired fasting glucose in the Malaysian population. When the probability of an observed variation occurring by random chance was less than 5%, it was deemed statistically significant, according to a significance level of p <  0.05.

## Results

### Socio-demographic characteristics

In all, 6,183 people took part in the research. Of these, 2,444 respondents, or 39.5%, were from rural areas, and 3,739 respondents, or 60.5%, lived in metropolitan areas. The distribution of respondents by gender was almost equal, with slightly more women (54%, n =  3,341) than men (46%, n =  2,842). The age range of the respondents was vast, with 47.4% (n = 2,928) being between the ages of 18 and 39, 32.4% (n = 2,008) being between the ages of 40 and 59, and 20.2% (n = 1,247) being over the age of 60. The majority of respondents (63.7%), followed by Others (19.9%), Chinese (11.5%), and Indian (4.9%), identified as Malay. Married people made up 65.3% of the sample, which was the biggest percentage of respondents.

Respondents’ educational backgrounds ranged widely: 47.5% had completed secondary school, 27.8% had just completed elementary school, and 24.7% had completed tertiary education. Approximately 67.3% of respondents reported a low-income status (Bottom 40), while 24.3% fell into the middle-income bracket (Middle 40), and 8.4% were classified as high-income earners or Top20 (Table 1). For household income group characteristics, B40 households belong to the lowest income group, representing the bottom 40% of earners in Malaysia. The middle-income group (Middle 40% or M40) comprises the next 40% of households, while T20 represents the highest-income households, making up the top 20% of earners [17]. The socio-demographic characteristics of the study population are presented in Table 1.

**Table 1:** Socio-demographic characteristics.

Characteristic	n	%
Locality		
Urban	3739	60.5
Rural	2444	39.5
Sex		
Male	2842	46.0
Female	3341	54.0
Age Group		
18–39	2928	47.4
40–59	2008	32.4
≥ 60	1247	20.2
Ethnicity		
Malay	3938	63.7
Chinese	710	11.5
Indian	302	4.9
Others	1233	19.9
Marital status		
Unmarried	2148	34.7
Married	4035	65.3
Educational status		
No education/ Primary Education	1717	27.8
Secondary Education	2929	47.5
Tertiary Education	1522	24.7
Household Income Group		
Bottom 40	3944	67.3
Middle 40	1422	24.3
Top 20	495	8.4

* The socio-demographic characteristics of the study population are presented in S1 Table in S1 File.

### Prevalence of impaired fasting glucose

The prevalence of impaired fasting glucose (IFG) across the study population was found to be 22.6%. Significant variances in IFG prevalence were observed across various demographic and health-related factors, including age group (p <  0.001), marital status (p <  0.001), Body Mass Index (BMI) (p <  0.001), hypertensive status (p =  0.002) and hypercholesterolemia status (p =  0.001), as shown in Table 2.

**Table 2:** Prevalence of impaired fasting glucose (IFG) by socio-demographic characteristics.

Characteristic/ Category	Not impaired			IFG			P value
	Unweighted count	Estimated population	Prevalence % (95% CI)	Unweighted count (n)	Estimated population	Prevalence % (95% CI)	
Total	4608	9633620	77.4 (75.1–79.6)	1575	2806830	22.6 (20.4–24.9)	
Locality							
Urban	2760	7164207	77.0 (74.1–79.6)	979	2143083	23.0 (20.4–25.3)	0.414
Rural	1848	2469412	78.8 (75.2–82.0)	596	663747	21.2 (18.0–24.8)	
Sex							
Male	2152	5012788	78.4 (75.4–81.0)	690	1384483	21.6 (19.0–24.6)	0.268
Female	2456	4620832	76.5 (73.5–79.2)	885	1422347	23.5 (20.8–26.5)	
Age Group							
18–39	2325	6131560	80.5 (77.6–83.1)	603	1485971	19.5 (16.9–22.4)	p < 0.001
40–59	1417	2459723	73.9 (70.4–77.1)	591	868487	26.1 (22.9–29.6)	
≥ 60	866	1042336	69.7 (65.6–73.6)	381	452371	30.2 (26.4–34.4)	
Ethnicity							
Malay	2857	4831668	75.6 (72.7–78.3)	1081	1557929	24.4 (21.7–27.3)	0.015
Chinese	530	1725797	74.4 (67.9–80.0)	180	594387	25.6 (20.0–32.1)	
Indian	223	383177	77.4 (67.6–85.0)	79	111697	22.6 (15.0–32.4)	
Others	998	2692976	83.2 (78.9–86.8)	235	542817	16.8 (13.2–22.1)	
Marital status							
Unmarried	1691	4085009	82.1 (79.3–84.6)	457	889241	17.9 (15.4–20.7)	p < 0.001
Married	2917	5548611	74.3 (71.3–77.1)	1118	1917589	25.7 (22.9–28.7)	
Educational status							
No education/ Primary Education	1262	2356204	78.0 (73.9–81.6)	445	665588	22.0 (18.4–26.1)	0.071
Secondary Education	2182	4602436	75.5 (72.1–78.6)	747	1492648	24.5 (21.4–27.9)	
Tertiary Education	1151	2638546	80.5 (77.2–83.4)	371	639096	19.5 (16.6–22.8)	
Household Income Group							
Bottom 40	2927	5857519	76.9 (73.8–79.6)	1017	1764253	23.1 (20.4–26.2)	0.215
Middle 40	1056	2426757	79.2 (75.5–82.4)	366	637797	20.8 (17.6–24.5)	
Top 20	385	924659	81.7 (75.8–86.4)	110	207694	18.3 (13.6–24.2)	
BMI							
Underweight	315	747034	82.0 (75.4–87.1)	80	164150	18.0 (12.9–24.6)	p < 0.001
Normal	1949	4461010	80.6 (77.6–83.2)	596	1075089	19.4 (16.8–22.4)	
Overweight/ Obese	2218	4246615	74.3 (71.3–77.0)	850	1472504	25.7 (23.0–28.7)	
Hypertension							
No	3839	8510777	78.1 (75.6–80.5)	1238	2380483	21.9 (19.5–24.4)	0.002
Yes	767	1113284	72.3 (68.6–75.7)	337	426347	27.7 (24.3–31.4)	
Hypercholesterolemia							
No	3959	8705506	78.2 (75.7–80.5)	1276	2423655	21.8 (19.5–24.3)	0.001
Yes	649	928114	70.8 (66.4–74.8)	299	383175	29.2 (25.2–33.6)	
Physical Activity Level							
Inactive	1081	2278457	78.2 (74.1–81.9)	374	633984	21.8 (18.1–25.9)	0.598
Active	3480	7251011	77.2 (74.7–79.5)	1190	2146543	22.8 (20.5–25.3)	
Current Smokers							
No	3628	7281735	77.0 (74.6–79.2)	1277	2180291	23.0 (20.8–25.4)	0.321
Yes	965	2319157	79.0 (74.6–82.9)	295	614907	21.0 (17.1–25.4)	

* The prevalence of impaired fasting glucose (IFG) across different socio-demographic groups is presented in S2 Table in S1 File.

### Factors associated with impaired fasting glucose

A multivariate analysis using multiple logistic regression was conducted to identify factors associated with impaired fasting glucose. Significant associations between impaired fasting glucose and certain demographic factors were found by the analysis: Individuals aged 60 years and above exhibited a higher likelihood of impaired fasting glucose (Adjusted Odds Ratio [AOR] 1.51, 95% Confidence Interval [CI]: 1.01, 2.06). The adjusted odds ratio (AOR) was 1.51, indicating that older individuals have a 51% higher likelihood of impaired fasting glucose. The 95% confidence interval (CI) ranged from 1.01 to 2.06, suggesting a statistically significant association (p <  0.05). Similarly, married individuals were found to have a higher likelihood of impaired fasting glucose, with The AOR was 1.46, with 95% CI: 1.16, 1.84), indicating a 46% higher likelihood compared to unmarried individuals, which is statistically significant (p <  0.05). However, no statistically significant associations were found between impaired fasting glucose and other factors (locality, gender, educational level, household income, BMI, Hypertensive status, hypercholesterolemia status, physical activities, and smoking status). The p-values for these variables were above the 0.05 significance threshold, indicating no strong evidence of an association in this sample.

## Discussion

Research continues to investigate how IFG relates to the risk of developing diabetes, other cardiovascular diseases, as well as mortality. A 2017 longitudinal study of 1167 women aged 20–94 years in the Geelong Osteoporosis Study found that while diabetes was associated with increased all-cause mortality, IFG showed no such association over a 10-year period in Australian women [18]. However, a multicenter retrospective cohort study of 312 hospitalized COVID-19 patients from five Wuhan hospitals reported that both IFG and diabetes at admission were associated with higher risks of adverse outcomes in COVID-19 patients [19]. Additionally, a survey among 3,615 Shinawatra employees identified higher coronary risk factors, including blood pressure level, BMI, and serum lipids, in individuals with IFG and DM compared to those with normal glucose levels [20].

Our study revealed that 22% of Malaysia’s population without diabetes is at risk of developing the disease and other cardiovascular conditions, with prevalence rates ranging from 22.6%. This is supported by several studies. A cross-sectional study was done in Diabetic Clinic in Shaikh Zayed Hospital from July to December 2017 resulting in a prevalence of 31% of IFG, with a range of fasting glucose of 5.6 – 7.0mmol/L [21]. In India, a cross-sectional study conducted in 2017 and 2018 on 12000 households revealed that 24.5% of respondents had impaired fasting glucose readings [22].

### Age-related findings

The prevalence of impaired fasting glucose (IFG) increases with age, with the highest prevalence observed among individuals aged 60 years and older, as shown in Table 2. Our multivariate analysis (Table 3) revealed that individuals in this age group were 1.51 times more likely to have IFG compared to younger age groups (Adjusted Odds Ratio [AOR] 1.51, 95% CI: 1.01–2.06). This relationship may be attributed to age-related physiological changes, such as reduced insulin production and decreased hepatic sensitivity to insulin’s activity in regulating glucose output [23]. These factors contribute to impaired glucose metabolism, making older adults more susceptible to glucose dysregulation. Supporting evidence for this age-related trend can be found in a cross-sectional study conducted among 15,603 non-diabetic individuals in Hong Kong. The study reported a significant increase in plasma glucose levels, including fasting plasma glucose, random plasma glucose, and 2-hour post-prandial plasma glucose, with advancing age [24]. This aligns with our findings and underscores the strong link between aging and impaired glucose regulation. The observed association between age and IFG highlights the need for targeted screening and intervention strategies for older populations. Given the physiological vulnerabilities associated with aging, early detection of IFG is critical to prevent progression to diabetes and associated complications. Future research should explore additional factors that may interact with age, such as lifestyle, comorbidities, and genetic predispositions, to further elucidate the mechanisms underlying this relationship.

**Table 3:** Factors associated with impaired fasting glycemia among the Malaysian Adult Population (n = 6183).

Characteristic/ Category	OR (95% CI)	P value	aOR (95% CI)	P value
Locality				
Urban	1.11 (0.86, 1.44)	0.414	1.06 (0.80–1.40)	0.692
Rural	1.00 (ref)		1.00 (ref)	
Sex				
Male	0.89 (0.74,1.09)	0.268	0.99 (0.79, 1.25)	0.96
Female	1.00 (ref)		1.00 (ref)	
Age Group				
18–39	1.00 (ref)		1.00 (ref)	
40–59	1.46 (1.19, 1.79)	p < 0.001	1.17 (0.93, 1.47)	0.175
≥ 60	1.79 (1.41, 2.27)	p < 0.001	1.51 (1.01, 2.06)	0.011
Ethnicity				
Malay	1.11 (0.66, 1.86)	0.702	1.13 (0.66, 1.94)	0.656
Chinese	1.18 (0.67, 2.10)	0.569	0.99 (0.55, 1.81)	0.989
Indian	0.69 (0.39, 1.21)	0.195	0.66 (0.38, 1.17)	0.158
Others	1.00 (ref)		1.00 (ref)	
Marital status				
Unmarried	1.00 (ref)		1.00 (ref)	
Married	1.58 (1.30, 1.95)	p < 0.001	1.46 (1.16, 1.84)	0.001
Educational status				
No education/ Primary Education	1.16 (0.86, 1.57)	0.311	1.04 (0.73, 1.48)	0.828
Secondary Education	1.34 (1.05, 1.70)	0.018	1.15 (0.89, 1.49)	0.279
Tertiary Education	1.00 (ref)		1.00 (ref)	
Household Income Group				
Bottom 40	1.34 (0.92, 1.97)	0.134	1.34 (0.89, 2.04)	0.165
Middle 40	1.00 (ref)		1.00 (ref)	
Top 20	1.17 (0.78, 1.75)	0.444	1.16 (0.75, 1.79)	0.510
BMI				
Underweight	1.00 (ref)		1.00 (ref)	
Normal	1.09 (0.74, 1.61)	0.637	0.88 (0.60, 1.33)	0.562
Overweight/ Obese	1.58 (1.05, 2.36)	0.027	1.24(0.81, 1.89)	0.316
Hypertension				
No	1.00 (ref)		1.00 (ref)	
Yes	1.37 (1.12, 1.67)	0.002	0.89 (0.69, 1.17)	0.411
Hypercholesterolemia				
No	1.00 (ref)		1.00 (ref)	
Yes	1.43 (1.18, 1.86)	p < 0.001	1.31 (0.97, 1.78)	0.082
Physical Activity Level				
Inactive	1.00 (ref)		1.00 (ref)	
Active	1.06 (0.85, 1.34)	0.598	1.13 (0.88,1.47)	0.340
Current Smokers				
No	1.00 (ref)		1.00 (ref)	
Yes	0.87 (0.69, 1.13)	0.321	0.96 (0.73, 1.26)	0.748

* p < 0.05 was considered statistically significant.

** The factors associated with IFG are presented in S3 Table in S1 File.

### Weight and IFG

Our study found a significant association between Body Mass Index (BMI) and impaired fasting glucose (IFG) (p <  0.001), as shown in Table 2. Interestingly, the highest prevalence of IFG was observed among individuals classified as overweight or obese, followed by those with normal BMI, and the lowest prevalence was found among underweight individuals. This distribution aligns with previous studies that have reported a strong association between excess body weight and the risk of IFG or diabetes. A Chinese study tracking individuals from the 2010 China Chronic Disease and Risk Factor Surveillance, which examined the association between BMI and the risk of IGT and IFG in Chinese individuals, found that being overweight and obese increased the risk of developing IFG [25]. Similarly, a cross-sectional study in Chile reported a higher prevalence of IFG among overweight and obese individuals [26]. However, it is noteworthy that even underweight individuals in our study exhibited a measurable prevalence of IFG (18.0%), challenging the traditional view that IFG is primarily linked to excess body weight. While the prevalence among the underweight group is lower than in those with overweight or obesity, it remains clinically significant. Other research has also highlighted this phenomenon. For example, a study involving adult men of varying weight showed that moderate underweight is associated with impaired glucose tolerance (IGT) in healthy human volunteers [27]. Another Japanese study reported higher IGT prevalence among underweight women (13.3%) than those with normal weight (1.8%), citing impaired early-phase insulin secretion, low fitness levels, and reduced insulin sensitivity as possible factors [28]. These observations underscore the complexity of the relationship between BMI and IFG. While overweight and obese individuals are at a higher risk of developing IFG due to insulin resistance, underweight individuals may also experience glucose regulation abnormalities due to different physiological mechanisms. This highlights the importance of not limiting diabetes screening to individuals with excess body weight. Screening programs should include underweight individuals, particularly those with other risk factors, to ensure early detection and prevention of progression to diabetes.

### Hypercholesterolemia and hypertension

Our findings indicated a higher prevalence of IFG among respondents with hypercholesterolemia and hypertension, as shown in Table 2. Similar associations have been observed in other studies. A cross-sectional population-based study in Chile found a significant correlation between IFG and older age, a family history of diabetes, lower educational attainment, overweight, obesity, and central obesity, as well as hypertension, hypercholesterolemia, and hypertriglyceridemia [26]. A study in Sri Lanka reported a significant association between high BMI, waist circumference, blood pressure, and hypercholesterolemia with IFG [29]. The use of statin, which has been associated with adverse effects on insulin sensitivity and the emergence of new-onset diabetes might contribute to this relationship. A cross-sectional, analytical study in Islamabad reported a 42.6% prevalence among respondents with hypertension, suggesting the need of early IFG screening [30]. In a different study involving 1026 essential hypertensive outpatients in China reported a prevalence of 30.5% IFG among respondents [31]. A Japanese study revealed a link between hypertension and isolated IFG and normal fasting glucose, emphasizing that the prevalence of hypertension increases with worsening glucose metabolism [32]. Results in Table 2 suggest that patients with hypertension and hypercholesterolemia may benefit from diabetes screening. The risk of having IFG in a person with known hypertension and hypercholesterolemia, however, was not significant, according to Table 3, which indicated no significant correlation between it.

### Marital status and IFG

Our multivariate analysis indicated a significant association between being married and having IFG, with married individuals being 1.4 times more likely to develop IFG than single people. This finding aligns with NHMS 2015 data. However, global studies on the association between marital status and IFG presents mixed results, reflecting the complexity of this association. For example, the Baependi Heart Study (BHS) conducted in Brazil examined risk factors for cardiovascular and other non-communicable diseases. While this study found that married individuals were more likely to have increased weight, they were less likely to develop type 2 diabetes, suggesting potential protective effects of marriage against diabetes progression [33]. Contrastingly, a national study in Laos found no significant correlation between marital status and IFG, highlighting regional and cultural differences in the association [34]. Another study conducted in Tehran investigated the incidence of pre-diabetes and associated risk factors among 5879 individuals aged ≥ 20 years who were free of diabetes and pre-diabetes. The findings showed a higher prevalence of IFG among unmarried women, with the researchers attributing lower diabetes incidence among married women to reduced stress and the adoption of healthier behaviors. These behaviors might include better dietary habits and more consistent mealtimes, which are commonly observed in family settings. Hadaegh et al. (2017) reported a higher prevalence of IFG among unmarried women in their **9-year follow-up study on prediabetes incidence and risk factors**. The researchers suggested that the lower diabetes incidence observed among married women could be attributed to **reduced stress levels and the adoption of healthier lifestyle behaviors**, such as improved dietary habits and more consistent mealtime routines, which are commonly observed in family settings [35].

The observed relationship between marital status and IFG in our study may be influenced by various lifestyle factors. Married individuals often have different food preferences, food preparation practices, and more regular mealtime routines, which could impact glucose metabolism. However, the stress of maintaining a family, combined with **limited time for physical activity** and **higher caloric intake** in family meals, may increase IFG risk among married individuals. **Chen et al. (2024)** found that marriage was associated with a higher risk of prediabetes among healthcare workers, with **triglyceride levels mediating this relationship**, highlighting the complex metabolic effects of marital status [36]. **Similarly, Monsivais et al. (2014)** reported that **greater time spent on home food preparation was linked to healthier eating habits**, suggesting that time constraints may lead to greater reliance on **less nutritious, convenience foods**, which could negatively affect glucose metabolism [37]. These findings underscore the importance of considering socio-cultural and behavioral factors when interpreting the relationship between marital status and metabolic health. In summary, while our study adds to the body of evidence linking marital status to IFG, the association is likely multifactorial and context-dependent. Further longitudinal research is needed to clarify the mechanisms underlying this relationship and to explore how marital dynamics, stress, and lifestyle factors collectively influence glucose metabolism.

### Limitations

This study has several limitations that should be considered when interpreting the findings. First, the use of finger-prick blood samples instead of fasting plasma samples may result in variations in glucose measurements, potentially affecting the accuracy of IFG classification.

Second, fasting glucose analysis in this study did not utilize continuous data, which may limit the ability to detect subtle variations in glucose levels and associations with risk factors. Third, the study lacked data on certain key risk factors for undiagnosed diabetes mellitus, such as family history of diabetes and history of gestational diabetes, which may limit the identification of significant associations.

Furthermore, the reliance on self-reported data introduces the possibility of recall bias and underreporting, particularly among individuals with undiagnosed diabetes who may not be aware of their condition. Furthermore, the use of different criteria and cut-off points for defining IFG in other studies could account for discrepancies in results and the number of associated factors identified.

To enhance the comprehensiveness of diabetes screening, future studies should consider combining the oral glucose tolerance test (OGTT) with IFG assessments to capture a broader spectrum of glucose dysregulation [38]. Lastly, the cross-sectional design of this study limits the ability to draw causal inferences. Longitudinal studies are recommended to explore the temporal relationships between risk factors and IFG, thereby informing the development of more effective preventive strategies and interventions.

By addressing these limitations, future research could provide a more robust understanding of the factors contributing to impaired fasting glucose and its progression to diabetes.

## Conclusion

The rising prevalence of impaired fasting glucose (IFG) is a growing public health concern due to its link with diabetes and cardiovascular disease. Screening programs should consider factors such as age, marital status, and co-morbidities like hypertension and hypercholesterolemia, while also addressing the risk among underweight individuals. Expanding screening beyond traditional at-risk groups and ensuring early detection are vital for prevention. Future research should explore the mechanisms underlying these associations to improve screening and intervention strategies. Additionally, adopting standardized IFG cut-off points will enhance consistency in identifying risk factors and support efforts to reduce diabetes-related complications.

## Supporting information

S1 File **S1 Table1. Frequency Table Socio-demographic characteristics.** This table provides a breakdown of the socio-demographic data used in the study. **S2 Table. Prevalence of impaired fasting glucose (IFG) by socio-demographic characteristics.** This table presents the prevalence of impaired fasting glucose (IFG) across different socio-demographic groups, highlighting variations based on age, sex, ethnicity, marital status, education level, employment status, and household income category. The following sub-tables (S2 Table 1 to S2 Table 14) provide a detailed breakdown: **S2 Table 1: Prevalence of impaired fasting glucose (IFG) by socio-demographic characteristics (Total). S2 Table 2: Prevalence of impaired fasting glucose (IFG) by socio-demographic characteristics (Locality). S2 Table 3: Prevalence of impaired fasting glucose (IFG) by socio-demographic characteristics (Sex). S2 Table 4: Prevalence of impaired fasting glucose (IFG) by socio-demographic characteristics (Age Group). S2 Table 5: Prevalence of impaired fasting glucose (IFG) by socio-demographic characteristics (Ethnicity). S2 Table 6: Prevalence of impaired fasting glucose (IFG) by socio-demographic characteristics (Marital Status). S2 Table 7: Prevalence of impaired fasting glucose (IFG) by socio-demographic characteristics (Educational Status). S2 Table 8: Prevalence of impaired fasting glucose (IFG) by socio-demographic characteristics (Household Income Group). S2 Table 9: Prevalence of impaired fasting glucose (IFG) by socio-demographic characteristics (BMI). S2 Table 10: Prevalence of impaired fasting glucose (IFG) by socio-demographic characteristics (Hypertension). S2 Table 11: Prevalence of impaired fasting glucose (IFG) by socio-demographic characteristics (Hypercholesterolemia). S2 Table 12: Prevalence of impaired fasting glucose (IFG) by socio-demographic characteristics (Physical Activity Level). S2 Table 13: Prevalence of impaired fasting glucose (IFG) by socio-demographic characteristics (Current Smokers). S2 Table 14: Prevalence of impaired fasting glucose (IFG) by socio-demographic characteristics (P value). S3 Table. Factors associated with impaired fasting glucose (IFG) among the Malaysian adult population.** This table presents the results of the logistic regression analysis identifying factors associated with IFG, including socio-demographic characteristics, lifestyle behaviors, and health-related variables. Odds ratios (OR), Adjusted odds ratios (aOR) and confidence intervals (CI) are provided to indicate the strength of associations. The following sub-tables (S3 Table 1 to S3 Table 13) provide a detailed breakdown: **S3 Table 1: Factors associated with Impaired Fasting Glycemia Among the Malaysian Adult Population (Locality: OR(95%CI). S3 Table 2: Factors associated with Impaired Fasting Glycemia Among the Malaysian Adult Population (Sex: OR(95%CI). S3 Table 3: Factors associated with Impaired Fasting Glycemia Among the Malaysian Adult Population (Age group: OR(95%CI). S3 Table 4: Factors associated with Impaired Fasting Glycemia Among the Malaysian Adult Population (Ethnicity: OR(95%CI). S3 Table 5: Factors associated with Impaired Fasting Glycemia Among the Malaysian Adult Population (Marital Status: OR(95%CI). S3 Table 6: Factors associated with Impaired Fasting Glycemia Among the Malaysian Adult Population (Educational Status: OR(95%CI). S3 Table 7: Factors associated with Impaired Fasting Glycemia Among the Malaysian Adult Population (Household Income: OR(95%CI). S3 Table 8: Factors associated with Impaired Fasting Glycemia Among the Malaysian Adult Population (BMI: OR(95%CI). S3 Table 9: Factors associated with Impaired Fasting Glycemia Among the Malaysian Adult Population (Hypertension: OR(95%CI). S3 Table 10: Factors associated with Impaired Fasting Glycemia Among the Malaysian Adult Population (Hypercholesterolemia: OR(95%CI). S3 Table 11: Factors associated with Impaired Fasting Glycemia Among the Malaysian Adult Population (Physical Activity Level: OR(95%CI). S3 Table 12: Factors associated with Impaired Fasting Glycemia Among the Malaysian Adult Population (Current Smokers: OR(95%CI). S3 Table 13: Factors associated with Impaired Fasting Glycemia Among the Malaysian Adult Population (aOR(95%CI).**
(DOCX)

## Abbreviations

IFG: Impaired Fasting Glucose
T2DM: Type 2 Diabetes Mellitus
NHMS: National Health & Morbidity Survey
WHO: World Health Organization
FBG: Fasting Blood Glucose
IGT: Impaired Glucose Tolerance
OGTT: Oral Glucose Tolerance Test
MI: Myocardial Infarction
BMI: Body Mass Index
DM: Diabetes Mellitus.
