# Supplementary material for: Prevalence of impaired fasting glucose and associated risk factors among Malaysian adult population: Findings from the National Health and Morbidity Survey (NHMS) 2019
Source: PLoS One. 2025 Apr 16;20(4):e0320993. doi: 10.1371/journal.pone.0320993 (PMC12002514; doi:10.1371/journal.pone.0320993)
Supplement: S1 File — S1 Table1. Frequency Table Socio-demographic characteristics. This table provides a breakdown of the socio-demographic data used in the study. S2 Table. Prevalence of impaired fasting glucose (IFG) by socio-demographic characteristics. This table presents the prevalence of impaired fasting glucose (IFG) across different socio-demographic groups, highlighting variations based on age, sex, ethnicity, marital status, education level, employment status, and household income category. The following sub-tables (S2 Table 1 to S2 Table 14) provide a detailed breakdown: S2 Table 1: Prevalence of impaired fasting glucose (IFG) by socio-demographic characteristics (Total). S2 Table 2: Prevalence of impaired fasting glucose (IFG) by socio-demographic characteristics (Locality). S2 Table 3: Prevalence of impaired fasting glucose (IFG) by socio-demographic characteristics (Sex). S2 Table 4: Prevalence of impaired fasting glucose (IFG) by socio-demographic characteristics (Age Group). S2 Table 5: Prevalence of impaired fasting glucose (IFG) by socio-demographic characteristics (Ethnicity). S2 Table 6: Prevalence of impaired fasting glucose (IFG) by socio-demographic characteristics (Marital Status). S2 Table 7: Prevalence of impaired fasting glucose (IFG) by socio-demographic characteristics (Educational Status). S2 Table 8: Prevalence of impaired fasting glucose (IFG) by socio-demographic characteristics (Household Income Group). S2 Table 9: Prevalence of impaired fasting glucose (IFG) by socio-demographic characteristics (BMI). S2 Table 10: Prevalence of impaired fasting glucose (IFG) by socio-demographic characteristics (Hypertension). S2 Table 11: Prevalence of impaired fasting glucose (IFG) by socio-demographic characteristics (Hypercholesterolemia). S2 Table 12: Prevalence of impaired fasting glucose (IFG) by socio-demographic characteristics (Physical Activity Level). S2 Table 13: Prevalence of impaired fasting glucose (IFG) by socio-demographic characteristics (Current Smo [file pone.0320993.s001.docx]

**S1 Table1. Frequency Table Socio-demographic characteristics**

- This table corresponds to Table 1 in the manuscript

| **[Final] Locality** | | | | | |
| --- | --- | --- | --- | --- | --- |
|  | | Frequency | Percent | Valid Percent | Cumulative Percent |
| Valid | Urban | 3739 | 60.5 | 60.5 | 60.5 |
|  | Rural | 2444 | 39.5 | 39.5 | 100.0 |
|  | Total | 6183 | 100.0 | 100.0 |  |
|  |  |  |  |  |  |
| **[Final] Gender** | | | | | |
|  | | Frequency | Percent | Valid Percent | Cumulative Percent |
| Valid | Male | 2842 | 46.0 | 46.0 | 46.0 |
|  | Female | 3341 | 54.0 | 54.0 | 100.0 |
|  | Total | 6183 | 100.0 | 100.0 |  |
|  |  |  |  |  |  |
| **Age Recode 3 group** | | | | | |
|  | | Frequency | Percent | Valid Percent | Cumulative Percent |
| Valid | 18-39 | 2928 | 47.4 | 47.4 | 47.4 |
|  | 40-59 | 2008 | 32.5 | 32.5 | 79.8 |
|  | >=60 | 1247 | 20.2 | 20.2 | 100.0 |
|  | Total | 6183 | 100.0 | 100.0 |  |
|  |  |  |  |  |  |
| **Marital Status 2group** | | | | | |
|  | | Frequency | Percent | Valid Percent | Cumulative Percent |
| Valid | Single/Divorce/Widow | 2148 | 34.7 | 34.7 | 34.7 |
|  | Married | 4035 | 65.3 | 65.3 | 100.0 |
|  | Total | 6183 | 100.0 | 100.0 |  |
|  |  |  |  |  |  |
| **Race Recode 4 grp** | | | | | |
|  | | Frequency | Percent | Valid Percent | Cumulative Percent |
| Valid | Malay | 3938 | 63.7 | 63.7 | 63.7 |
|  | Chinese | 710 | 11.5 | 11.5 | 75.2 |
|  | Indian | 302 | 4.9 | 4.9 | 80.1 |
|  | Others | 1233 | 19.9 | 19.9 | 100.0 |
|  | Total | 6183 | 100.0 | 100.0 |  |
|  |  |  |  |  |  |
| **Education Recode 3 Group** | | | | | |
|  | | Frequency | Percent | Valid Percent | Cumulative Percent |
| Valid | No education/Primary | 1717 | 27.8 | 27.8 | 27.8 |
|  | Secondary | 2929 | 47.4 | 47.5 | 75.3 |
|  | Tertiery | 1522 | 24.6 | 24.7 | 100.0 |
|  | Total | 6168 | 99.8 | 100.0 |  |
| Missing | System | 15 | 0.2 |  |  |
| Total | | 6183 | 100.0 |  |  |
|  |  |  |  |  |  |
| **[Final] Household Income - By state cut off, DOSM 2016** | | | | | |
|  | | Frequency | Percent | Valid Percent | Cumulative Percent |
| Valid | Bottom 40% | 3944 | 63.8 | 67.3 | 67.3 |
|  | Middle 40% | 1422 | 23.0 | 24.3 | 91.6 |
|  | Top 20% | 495 | 8.0 | 8.4 | 100.0 |
|  | Total | 5861 | 94.8 | 100.0 |  |
| Missing | System | 322 | 5.2 |  |  |
| Total | | 6183 | 100.0 |  |  |

**S2 Table 1: Prevalence of impaired fasting glucose (IFG) by socio-demographic characteristics (Total)**

- All tables in S2 correspond to Table 2 in the manuscript

| **CPG_impaired (Total)** | | | | | | | |
| --- | --- | --- | --- | --- | --- | --- | --- |
|  | | Estimate | Standard Error | 95% Confidence Interval | | Coefficient of Variation | Unweighted Count |
|  |  |  |  | Lower | Upper |  |  |
| Population Size | no IFG | 9633620.573 | 293135.597 | 9057507.820 | 10209733.326 | 0.030 | 4608 |
|  | IFG 6.1-6.9 | 2806830.911 | 154492.058 | 2503200.626 | 3110461.195 | 0.055 | 1575 |
|  | Total | 12440451.484 | 315687.590 | 11820016.270 | 13060886.698 | 0.025 | 6183 |
| % of Total | no IFG | 77.4% | 1.1% | 75.1% | 79.6% | 0.015 | 4608 |
|  | IFG 6.1-6.9 | 22.6% | 1.1% | 20.4% | 24.9% | 0.051 | 1575 |
|  | Total | 100.0% | 0.0% | 100.0% | 100.0% | 0.000 | 6183 |

**S2 Table 2: Prevalence of impaired fasting glucose (IFG) by socio-demographic characteristics (Locality)**

|  | | | | | | |
| --- | --- | --- | --- | --- | --- | --- |
| [Final] Locality | | | | CPG_impaired | | |
|  |  |  |  | no IFG | IFG 6.1-6.9 | Total |
| Urban | Population Size | Estimate | | 7164207.801 | 2143083.082 | 9307290.883 |
|  |  | 95% Confidence Interval | Lower | 6638875.109 | 1860565.964 | 8738687.126 |
|  |  |  | Upper | 7689540.492 | 2425600.200 | 9875894.639 |
|  |  | Coefficient of Variation | | 0.037 | 0.067 | 0.031 |
|  |  | Unweighted Count | | 2760 | 979 | 3739 |
|  | % within [Final] Locality | Estimate | | 77.0% | 23.0% | 100.0% |
|  |  | 95% Confidence Interval | Lower | 74.1% | 20.4% | 100.0% |
|  |  |  | Upper | 79.6% | 25.9% | 100.0% |
|  |  | Coefficient of Variation | | 0.018 | 0.062 | 0.000 |
|  |  | Unweighted Count | | 2760 | 979 | 3739 |
| Rural | Population Size | Estimate | | 2469412.772 | 663747.829 | 3133160.601 |
|  |  | 95% Confidence Interval | Lower | 2232914.426 | 552502.928 | 2884907.458 |
|  |  |  | Upper | 2705911.118 | 774992.730 | 3381413.744 |
|  |  | Coefficient of Variation | | 0.049 | 0.085 | 0.040 |
|  |  | Unweighted Count | | 1848 | 596 | 2444 |
|  | % within [Final] Locality | Estimate | | 78.8% | 21.2% | 100.0% |
|  |  | 95% Confidence Interval | Lower | 75.2% | 18.0% | 100.0% |
|  |  |  | Upper | 82.0% | 24.8% | 100.0% |
|  |  | Coefficient of Variation | | 0.022 | 0.082 | 0.000 |
|  |  | Unweighted Count | | 1848 | 596 | 2444 |
| Total | Population Size | Estimate | | 9633620.573 | 2806830.911 | 12440451.484 |
|  |  | 95% Confidence Interval | Lower | 9057507.820 | 2503200.626 | 11820016.270 |
|  |  |  | Upper | 10209733.326 | 3110461.195 | 13060886.698 |
|  |  | Coefficient of Variation | | 0.030 | 0.055 | 0.025 |
|  |  | Unweighted Count | | 4608 | 1575 | 6183 |
|  | % within [Final] Locality | Estimate | | 77.4% | 22.6% | 100.0% |
|  |  | 95% Confidence Interval | Lower | 75.1% | 20.4% | 100.0% |
|  |  |  | Upper | 79.6% | 24.9% | 100.0% |
|  |  | Coefficient of Variation | | 0.015 | 0.051 | 0.000 |
|  |  | Unweighted Count | | 4608 | 1575 | 6183 |

**S2 Table 3: Prevalence of impaired fasting glucose (IFG) by socio-demographic characteristics (Sex)**

| **[Final] Gender * CPG_impaired** | | | | | | |
| --- | --- | --- | --- | --- | --- | --- |
| [Final] Gender | | | | CPG_impaired | | |
|  |  |  |  | no IFG | IFG 6.1-6.9 | Total |
| Male | Population Size | Estimate | | 5012788.430 | 1384483.411 | 6397271.842 |
|  |  | 95% Confidence Interval | Lower | 4612868.407 | 1191043.154 | 5961423.539 |
|  |  |  | Upper | 5412708.453 | 1577923.669 | 6833120.144 |
|  |  | Coefficient of Variation | | 0.041 | 0.071 | 0.035 |
|  |  | Unweighted Count | | 2152 | 690 | 2842 |
|  | % within [Final] Gender | Estimate | | 78.4% | 21.6% | 100.0% |
|  |  | 95% Confidence Interval | Lower | 75.4% | 19.0% | 100.0% |
|  |  |  | Upper | 81.0% | 24.6% | 100.0% |
|  |  | Coefficient of Variation | | 0.018 | 0.065 | 0.000 |
|  |  | Unweighted Count | | 2152 | 690 | 2842 |
| Female | Population Size | Estimate | | 4620832.143 | 1422347.499 | 6043179.642 |
|  |  | 95% Confidence Interval | Lower | 4302670.643 | 1232480.001 | 5694810.352 |
|  |  |  | Upper | 4938993.643 | 1612214.998 | 6391548.933 |
|  |  | Coefficient of Variation | | 0.035 | 0.068 | 0.029 |
|  |  | Unweighted Count | | 2456 | 885 | 3341 |
|  | % within [Final] Gender | Estimate | | 76.5% | 23.5% | 100.0% |
|  |  | 95% Confidence Interval | Lower | 73.5% | 20.8% | 100.0% |
|  |  |  | Upper | 79.2% | 26.5% | 100.0% |
|  |  | Coefficient of Variation | | 0.019 | 0.061 | 0.000 |
|  |  | Unweighted Count | | 2456 | 885 | 3341 |
| Total | Population Size | Estimate | | 9633620.573 | 2806830.911 | 12440451.484 |
|  |  | 95% Confidence Interval | Lower | 9057507.820 | 2503200.626 | 11820016.270 |
|  |  |  | Upper | 10209733.326 | 3110461.195 | 13060886.698 |
|  |  | Coefficient of Variation | | 0.030 | 0.055 | 0.025 |
|  |  | Unweighted Count | | 4608 | 1575 | 6183 |
|  | % within [Final] Gender | Estimate | | 77.4% | 22.6% | 100.0% |
|  |  | 95% Confidence Interval | Lower | 75.1% | 20.4% | 100.0% |
|  |  |  | Upper | 79.6% | 24.9% | 100.0% |
|  |  | Coefficient of Variation | | 0.015 | 0.051 | 0.000 |
|  |  | Unweighted Count | | 4608 | 1575 | 6183 |

**S2 Table 4: Prevalence of impaired fasting glucose (IFG) by socio-demographic characteristics (Age Group)**

| **Age Recode 3 group * CPG_impaired** | | | | | | |
| --- | --- | --- | --- | --- | --- | --- |
| Age Recode 3 group | | | | CPG_impaired | | |
|  |  |  |  | no IFG | IFG 6.1-6.9 | Total |
| 18-39 | Population Size | Estimate | | 6131560.067 | 1485971.144 | 7617531.211 |
|  |  | 95% Confidence Interval | Lower | 5664823.037 | 1257527.349 | 7106895.895 |
|  |  |  | Upper | 6598297.097 | 1714414.939 | 8128166.526 |
|  |  | Coefficient of Variation | | 0.039 | 0.078 | 0.034 |
|  |  | Unweighted Count | | 2325 | 603 | 2928 |
|  | % within Age Recode 3 group | Estimate | | 80.5% | 19.5% | 100.0% |
|  |  | 95% Confidence Interval | Lower | 77.6% | 16.9% | 100.0% |
|  |  |  | Upper | 83.1% | 22.4% | 100.0% |
|  |  | Coefficient of Variation | | 0.017 | 0.071 | 0.000 |
|  |  | Unweighted Count | | 2325 | 603 | 2928 |
| 40-59 | Population Size | Estimate | | 2459723.621 | 868487.959 | 3328211.580 |
|  |  | 95% Confidence Interval | Lower | 2269922.933 | 735314.984 | 3098235.126 |
|  |  |  | Upper | 2649524.308 | 1001660.935 | 3558188.034 |
|  |  | Coefficient of Variation | | 0.039 | 0.078 | 0.035 |
|  |  | Unweighted Count | | 1417 | 591 | 2008 |
|  | % within Age Recode 3 group | Estimate | | 73.9% | 26.1% | 100.0% |
|  |  | 95% Confidence Interval | Lower | 70.4% | 22.9% | 100.0% |
|  |  |  | Upper | 77.1% | 29.6% | 100.0% |
|  |  | Coefficient of Variation | | 0.023 | 0.065 | 0.000 |
|  |  | Unweighted Count | | 1417 | 591 | 2008 |
| >=60 | Population Size | Estimate | | 1042336.886 | 452371.808 | 1494708.693 |
|  |  | 95% Confidence Interval | Lower | 916980.965 | 376516.108 | 1337035.006 |
|  |  |  | Upper | 1167692.806 | 528227.508 | 1652382.381 |
|  |  | Coefficient of Variation | | 0.061 | 0.085 | 0.054 |
|  |  | Unweighted Count | | 866 | 381 | 1247 |
|  | % within Age Recode 3 group | Estimate | | 69.7% | 30.3% | 100.0% |
|  |  | 95% Confidence Interval | Lower | 65.6% | 26.4% | 100.0% |
|  |  |  | Upper | 73.6% | 34.4% | 100.0% |
|  |  | Coefficient of Variation | | 0.029 | 0.067 | 0.000 |
|  |  | Unweighted Count | | 866 | 381 | 1247 |
| Total | Population Size | Estimate | | 9633620.573 | 2806830.911 | 12440451.484 |
|  |  | 95% Confidence Interval | Lower | 9057507.820 | 2503200.626 | 11820016.270 |
|  |  |  | Upper | 10209733.326 | 3110461.195 | 13060886.698 |
|  |  | Coefficient of Variation | | 0.030 | 0.055 | 0.025 |
|  |  | Unweighted Count | | 4608 | 1575 | 6183 |
|  | % within Age Recode 3 group | Estimate | | 77.4% | 22.6% | 100.0% |
|  |  | 95% Confidence Interval | Lower | 75.1% | 20.4% | 100.0% |
|  |  |  | Upper | 79.6% | 24.9% | 100.0% |
|  |  | Coefficient of Variation | | 0.015 | 0.051 | 0.000 |
|  |  | Unweighted Count | | 4608 | 1575 | 6183 |

**S2 Table 5: Prevalence of impaired fasting glucose (IFG) by socio-demographic characteristics (Ethnicity)**

| **Race Recode 4 grp * CPG_impaired** | | | | | | |
| --- | --- | --- | --- | --- | --- | --- |
| Race Recode 4 grp | | | | CPG_impaired | | |
|  |  |  |  | no IFG | IFG 6.1-6.9 | Total |
| Malay | Population Size | Estimate | | 4831668.911 | 1557929.095 | 6389598.007 |
|  |  | 95% Confidence Interval | Lower | 4392500.434 | 1342188.459 | 5871010.054 |
|  |  |  | Upper | 5270837.389 | 1773669.731 | 6908185.959 |
|  |  | Coefficient of Variation | | 0.046 | 0.070 | 0.041 |
|  |  | Unweighted Count | | 2857 | 1081 | 3938 |
|  | % within Race Recode 4 grp | Estimate | | 75.6% | 24.4% | 100.0% |
|  |  | 95% Confidence Interval | Lower | 72.7% | 21.7% | 100.0% |
|  |  |  | Upper | 78.3% | 27.3% | 100.0% |
|  |  | Coefficient of Variation | | 0.019 | 0.059 | 0.000 |
|  |  | Unweighted Count | | 2857 | 1081 | 3938 |
| Chinese | Population Size | Estimate | | 1725797.811 | 594387.210 | 2320185.021 |
|  |  | 95% Confidence Interval | Lower | 1323460.410 | 426088.496 | 1846808.225 |
|  |  |  | Upper | 2128135.212 | 762685.924 | 2793561.817 |
|  |  | Coefficient of Variation | | 0.119 | 0.144 | 0.104 |
|  |  | Unweighted Count | | 530 | 180 | 710 |
|  | % within Race Recode 4 grp | Estimate | | 74.4% | 25.6% | 100.0% |
|  |  | 95% Confidence Interval | Lower | 67.9% | 20.0% | 100.0% |
|  |  |  | Upper | 80.0% | 32.1% | 100.0% |
|  |  | Coefficient of Variation | | 0.042 | 0.121 | 0.000 |
|  |  | Unweighted Count | | 530 | 180 | 710 |
| Indian | Population Size | Estimate | | 383177.586 | 111697.301 | 494874.886 |
|  |  | 95% Confidence Interval | Lower | 267417.302 | 62475.021 | 362908.400 |
|  |  |  | Upper | 498937.870 | 160919.580 | 626841.373 |
|  |  | Coefficient of Variation | | 0.154 | 0.224 | 0.136 |
|  |  | Unweighted Count | | 223 | 79 | 302 |
|  | % within Race Recode 4 grp | Estimate | | 77.4% | 22.6% | 100.0% |
|  |  | 95% Confidence Interval | Lower | 67.6% | 15.0% | 100.0% |
|  |  |  | Upper | 85.0% | 32.4% | 100.0% |
|  |  | Coefficient of Variation | | 0.057 | 0.196 | 0.000 |
|  |  | Unweighted Count | | 223 | 79 | 302 |
| Others | Population Size | Estimate | | 2692976.265 | 542817.305 | 3235793.570 |
|  |  | 95% Confidence Interval | Lower | 2314456.800 | 402141.967 | 2817504.790 |
|  |  |  | Upper | 3071495.730 | 683492.643 | 3654082.350 |
|  |  | Coefficient of Variation | | 0.072 | 0.132 | 0.066 |
|  |  | Unweighted Count | | 998 | 235 | 1233 |
|  | % within Race Recode 4 grp | Estimate | | 83.2% | 16.8% | 100.0% |
|  |  | 95% Confidence Interval | Lower | 78.9% | 13.2% | 100.0% |
|  |  |  | Upper | 86.8% | 21.1% | 100.0% |
|  |  | Coefficient of Variation | | 0.024 | 0.119 | 0.000 |
|  |  | Unweighted Count | | 998 | 235 | 1233 |
| Total | Population Size | Estimate | | 9633620.573 | 2806830.911 | 12440451.484 |
|  |  | 95% Confidence Interval | Lower | 9057507.820 | 2503200.626 | 11820016.270 |
|  |  |  | Upper | 10209733.326 | 3110461.195 | 13060886.698 |
|  |  | Coefficient of Variation | | 0.030 | 0.055 | 0.025 |
|  |  | Unweighted Count | | 4608 | 1575 | 6183 |
|  | % within Race Recode 4 grp | Estimate | | 77.4% | 22.6% | 100.0% |
|  |  | 95% Confidence Interval | Lower | 75.1% | 20.4% | 100.0% |
|  |  |  | Upper | 79.6% | 24.9% | 100.0% |
|  |  | Coefficient of Variation | | 0.015 | 0.051 | 0.000 |
|  |  | Unweighted Count | | 4608 | 1575 | 6183 |

**S2 Table 6: Prevalence of impaired fasting glucose (IFG) by socio-demographic characteristics (Marital Status)**

| **Marital Status 2group * CPG_impaired** | | | | | | |
| --- | --- | --- | --- | --- | --- | --- |
| Marital Status 2group | | | | CPG_impaired | | |
|  |  |  |  | no IFG | IFG 6.1-6.9 | Total |
| Single/Divorce/Widow | Population Size | Estimate | | 4085009.381 | 889241.463 | 4974250.844 |
|  |  | 95% Confidence Interval | Lower | 3719760.971 | 746026.231 | 4574371.698 |
|  |  |  | Upper | 4450257.791 | 1032456.695 | 5374129.991 |
|  |  | Coefficient of Variation | | 0.045 | 0.082 | 0.041 |
|  |  | Unweighted Count | | 1691 | 457 | 2148 |
|  | % within Marital Status 2group | Estimate | | 82.1% | 17.9% | 100.0% |
|  |  | 95% Confidence Interval | Lower | 79.3% | 15.4% | 100.0% |
|  |  |  | Upper | 84.6% | 20.7% | 100.0% |
|  |  | Coefficient of Variation | | 0.016 | 0.075 | 0.000 |
|  |  | Unweighted Count | | 1691 | 457 | 2148 |
| Married | Population Size | Estimate | | 5548611.192 | 1917589.447 | 7466200.640 |
|  |  | 95% Confidence Interval | Lower | 5154258.411 | 1676024.726 | 7024601.306 |
|  |  |  | Upper | 5942963.974 | 2159154.169 | 7907799.974 |
|  |  | Coefficient of Variation | | 0.036 | 0.064 | 0.030 |
|  |  | Unweighted Count | | 2917 | 1118 | 4035 |
|  | % within Marital Status 2group | Estimate | | 74.3% | 25.7% | 100.0% |
|  |  | 95% Confidence Interval | Lower | 71.3% | 22.9% | 100.0% |
|  |  |  | Upper | 77.1% | 28.7% | 100.0% |
|  |  | Coefficient of Variation | | 0.020 | 0.057 | 0.000 |
|  |  | Unweighted Count | | 2917 | 1118 | 4035 |
|  |  | 95% Confidence Interval | Lower | 9057507.820 | 2503200.626 | 11820016.270 |
|  |  |  | Upper | 10209733.326 | 3110461.195 | 13060886.698 |
|  |  | Coefficient of Variation | | 0.030 | 0.055 | 0.025 |
|  |  | Unweighted Count | | 4608 | 1575 | 6183 |
|  | % within Marital Status 2group | Estimate | | 77.4% | 22.6% | 100.0% |
|  |  | 95% Confidence Interval | Lower | 75.1% | 20.4% | 100.0% |
|  |  |  | Upper | 79.6% | 24.9% | 100.0% |
|  |  | Coefficient of Variation | | 0.015 | 0.051 | 0.000 |
|  |  | Unweighted Count | | 4608 | 1575 | 6183 |

**S2 Table 7: Prevalence of impaired fasting glucose (IFG) by socio-demographic characteristics (Educational Status)**

| **Education Recode 3 Group * CPG_impaired** | | | | | | |
| --- | --- | --- | --- | --- | --- | --- |
| Education Recode 3 Group | | | | CPG_impaired | | |
|  |  |  |  | no IFG | IFG 6.1-6.9 | Total |
| No education/Primary | Population Size | Estimate | | 2356204.230 | 665588.298 | 3021792.529 |
|  |  | 95% Confidence Interval | Lower | 2039256.532 | 544447.150 | 2678529.134 |
|  |  |  | Upper | 2673151.928 | 786729.447 | 3365055.924 |
|  |  | Coefficient of Variation | | 0.068 | 0.093 | 0.058 |
|  |  | Unweighted Count | | 1262 | 455 | 1717 |
|  | % within Education Recode 3 Group | Estimate | | 78.0% | 22.0% | 100.0% |
|  |  | 95% Confidence Interval | Lower | 73.9% | 18.4% | 100.0% |
|  |  |  | Upper | 81.6% | 26.1% | 100.0% |
|  |  | Coefficient of Variation | | 0.025 | 0.088 | 0.000 |
|  |  | Unweighted Count | | 1262 | 455 | 1717 |
| Secondary | Population Size | Estimate | | 4602436.034 | 1492648.203 | 6095084.237 |
|  |  | 95% Confidence Interval | Lower | 4234609.220 | 1262346.379 | 5670626.005 |
|  |  |  | Upper | 4970262.849 | 1722950.027 | 6519542.470 |
|  |  | Coefficient of Variation | | 0.041 | 0.079 | 0.035 |
|  |  | Unweighted Count | | 2182 | 747 | 2929 |
|  | % within Education Recode 3 Group | Estimate | | 75.5% | 24.5% | 100.0% |
|  |  | 95% Confidence Interval | Lower | 72.1% | 21.4% | 100.0% |
|  |  |  | Upper | 78.6% | 27.9% | 100.0% |
|  |  | Coefficient of Variation | | 0.022 | 0.068 | 0.000 |
|  |  | Unweighted Count | | 2182 | 747 | 2929 |
| Tertiery | Population Size | Estimate | | 2638546.408 | 639096.556 | 3277642.964 |
|  |  | 95% Confidence Interval | Lower | 2333585.787 | 534462.207 | 2953129.128 |
|  |  |  | Upper | 2943507.029 | 743730.906 | 3602156.800 |
|  |  | Coefficient of Variation | | 0.059 | 0.083 | 0.050 |
|  |  | Unweighted Count | | 1151 | 371 | 1522 |
|  | % within Education Recode 3 Group | Estimate | | 80.5% | 19.5% | 100.0% |
|  |  | 95% Confidence Interval | Lower | 77.2% | 16.6% | 100.0% |
|  |  |  | Upper | 83.4% | 22.8% | 100.0% |
|  |  | Coefficient of Variation | | 0.020 | 0.081 | 0.000 |
|  |  | Unweighted Count | | 1151 | 371 | 1522 |
| Total | Population Size | Estimate | | 9597186.672 | 2797333.058 | 12394519.730 |
|  |  | 95% Confidence Interval | Lower | 9025494.102 | 2494197.371 | 11781357.116 |
|  |  |  | Upper | 10168879.242 | 3100468.745 | 13007682.344 |
|  |  | Coefficient of Variation | | 0.030 | 0.055 | 0.025 |
|  |  | Unweighted Count | | 4595 | 1573 | 6168 |
|  | % within Education Recode 3 Group | Estimate | | 77.4% | 22.6% | 100.0% |
|  |  | 95% Confidence Interval | Lower | 75.1% | 20.4% | 100.0% |
|  |  |  | Upper | 79.6% | 24.9% | 100.0% |
|  |  | Coefficient of Variation | | 0.015 | 0.051 | 0.000 |
|  |  | Unweighted Count | | 4595 | 1573 | 6168 |

**S2 Table 8: Prevalence of impaired fasting glucose (IFG) by socio-demographic characteristics (Household Income Group)**

| **[Final] Household Income - By state cut off, DOSM 2016 * CPG_impaired** | | | | | | |
| --- | --- | --- | --- | --- | --- | --- |
| [Final] Household Income - By state cut off, DOSM 2016 | | | | CPG_impaired | | |
|  |  |  |  | no IFG | IFG 6.1-6.9 | Total |
| Bottom 40% | Population Size | Estimate | | 5857519.542 | 1764253.290 | 7621772.832 |
|  |  | 95% Confidence Interval | Lower | 5369339.363 | 1521988.830 | 7080515.904 |
|  |  |  | Upper | 6345699.721 | 2006517.750 | 8163029.760 |
|  |  | Coefficient of Variation | | 0.042 | 0.070 | 0.036 |
|  |  | Unweighted Count | | 2927 | 1017 | 3944 |
|  | % within [Final] Household Income - By state cut off, DOSM 2016 | Estimate | | 76.9% | 23.1% | 100.0% |
|  |  | 95% Confidence Interval | Lower | 73.8% | 20.4% | 100.0% |
|  |  |  | Upper | 79.6% | 26.2% | 100.0% |
|  |  | Coefficient of Variation | | 0.019 | 0.063 | 0.000 |
|  |  | Unweighted Count | | 2927 | 1017 | 3944 |
| Middle 40% | Population Size | Estimate | | 2426757.007 | 637797.590 | 3064554.597 |
|  |  | 95% Confidence Interval | Lower | 2089767.480 | 518581.284 | 2684680.639 |
|  |  |  | Upper | 2763746.534 | 757013.896 | 3444428.554 |
|  |  | Coefficient of Variation | | 0.071 | 0.095 | 0.063 |
|  |  | Unweighted Count | | 1056 | 366 | 1422 |
|  | % within [Final] Household Income - By state cut off, DOSM 2016 | Estimate | | 79.2% | 20.8% | 100.0% |
|  |  | 95% Confidence Interval | Lower | 75.5% | 17.6% | 100.0% |
|  |  |  | Upper | 82.4% | 24.5% | 100.0% |
|  |  | Coefficient of Variation | | 0.022 | 0.084 | 0.000 |
|  |  | Unweighted Count | | 1056 | 366 | 1422 |
| Top 20% | Population Size | Estimate | | 924659.299 | 207694.228 | 1132353.527 |
|  |  | 95% Confidence Interval | Lower | 703202.749 | 133701.786 | 875809.268 |
|  |  |  | Upper | 1146115.849 | 281686.670 | 1388897.787 |
|  |  | Coefficient of Variation | | 0.122 | 0.181 | 0.115 |
|  |  | Unweighted Count | | 385 | 110 | 495 |
|  | % within [Final] Household Income - By state cut off, DOSM 2016 | Estimate | | 81.7% | 18.3% | 100.0% |
|  |  | 95% Confidence Interval | Lower | 75.8% | 13.6% | 100.0% |
|  |  |  | Upper | 86.4% | 24.2% | 100.0% |
|  |  | Coefficient of Variation | | 0.033 | 0.147 | 0.000 |
|  |  | Unweighted Count | | 385 | 110 | 495 |
| Total | Population Size | Estimate | | 9208935.848 | 2609745.108 | 11818680.956 |
|  |  | 95% Confidence Interval | Lower | 8633764.707 | 2320022.725 | 11201011.306 |
|  |  |  | Upper | 9784106.989 | 2899467.492 | 12436350.607 |
|  |  | Coefficient of Variation | | 0.032 | 0.056 | 0.027 |
|  |  | Unweighted Count | | 4368 | 1493 | 5861 |
|  | % within [Final] Household Income - By state cut off, DOSM 2016 | Estimate | | 77.9% | 22.1% | 100.0% |
|  |  | 95% Confidence Interval | Lower | 75.6% | 19.9% | 100.0% |
|  |  |  | Upper | 80.1% | 24.4% | 100.0% |
|  |  | Coefficient of Variation | | 0.015 | 0.053 | 0.000 |
|  |  | Unweighted Count | | 4368 | 1493 | 5861 |

**S2 Table 9: Prevalence of impaired fasting glucose (IFG) by socio-demographic characteristics (BMI)**

| **BMI WHO 3 group * CPG_impaired** | | | | | | |
| --- | --- | --- | --- | --- | --- | --- |
| BMI WHO 3 group | | | | CPG_impaired | | |
|  |  |  |  | no IFG | IFG 6.1-6.9 | Total |
| underweight | Population Size | Estimate | | 747034.915 | 164150.866 | 911185.780 |
|  |  | 95% Confidence Interval | Lower | 616413.911 | 106224.850 | 768435.990 |
|  |  |  | Upper | 877655.918 | 222076.881 | 1053935.570 |
|  |  | Coefficient of Variation | | 0.089 | 0.180 | 0.080 |
|  |  | Unweighted Count | | 315 | 80 | 395 |
|  | % within BMI WHO 3 group | Estimate | | 82.0% | 18.0% | 100.0% |
|  |  | 95% Confidence Interval | Lower | 75.4% | 12.9% | 100.0% |
|  |  |  | Upper | 87.1% | 24.6% | 100.0% |
|  |  | Coefficient of Variation | | 0.036 | 0.164 | 0.000 |
|  |  | Unweighted Count | | 315 | 80 | 395 |
| normal weight | Population Size | Estimate | | 4461010.660 | 1075089.645 | 5536100.304 |
|  |  | 95% Confidence Interval | Lower | 4083675.515 | 900899.209 | 5110654.904 |
|  |  |  | Upper | 4838345.804 | 1249280.081 | 5961545.704 |
|  |  | Coefficient of Variation | | 0.043 | 0.082 | 0.039 |
|  |  | Unweighted Count | | 1949 | 596 | 2545 |
|  | % within BMI WHO 3 group | Estimate | | 80.6% | 19.4% | 100.0% |
|  |  | 95% Confidence Interval | Lower | 77.6% | 16.8% | 100.0% |
|  |  |  | Upper | 83.2% | 22.4% | 100.0% |
|  |  | Coefficient of Variation | | 0.018 | 0.073 | 0.000 |
|  |  | Unweighted Count | | 1949 | 596 | 2545 |
| overweight/obese | Population Size | Estimate | | 4246615.799 | 1472504.569 | 5719120.368 |
|  |  | 95% Confidence Interval | Lower | 3929340.365 | 1290289.967 | 5365042.119 |
|  |  |  | Upper | 4563891.232 | 1654719.171 | 6073198.617 |
|  |  | Coefficient of Variation | | 0.038 | 0.063 | 0.032 |
|  |  | Unweighted Count | | 2218 | 850 | 3068 |
|  | % within BMI WHO 3 group | Estimate | | 74.3% | 25.7% | 100.0% |
|  |  | 95% Confidence Interval | Lower | 71.3% | 23.0% | 100.0% |
|  |  |  | Upper | 77.0% | 28.7% | 100.0% |
|  |  | Coefficient of Variation | | 0.020 | 0.056 | 0.000 |
|  |  | Unweighted Count | | 2218 | 850 | 3068 |
| Total | Population Size | Estimate | | 9454661.373 | 2711745.079 | 12166406.452 |
|  |  | 95% Confidence Interval | Lower | 8883782.005 | 2417877.084 | 11550879.791 |
|  |  |  | Upper | 10025540.741 | 3005613.075 | 12781933.113 |
|  |  | Coefficient of Variation | | 0.031 | 0.055 | 0.026 |
|  |  | Unweighted Count | | 4482 | 1526 | 6008 |
|  | % within BMI WHO 3 group | Estimate | | 77.7% | 22.3% | 100.0% |
|  |  | 95% Confidence Interval | Lower | 75.4% | 20.1% | 100.0% |
|  |  |  | Upper | 79.9% | 24.6% | 100.0% |
|  |  | Coefficient of Variation | | 0.015 | 0.051 | 0.000 |
|  |  | Unweighted Count | | 4482 | 1526 | 6008 |

**S2 Table 10: Prevalence of impaired fasting glucose (IFG) by socio-demographic characteristics (Hypertension)**

| **[Final] Known Hypertension * CPG_impaired** | | | | | | |
| --- | --- | --- | --- | --- | --- | --- |
| [Final] Known Hypertension | | | | CPG_impaired | | |
|  |  |  |  | no IFG | IFG 6.1-6.9 | Total |
| No | Population Size | Estimate | | 8510777.529 | 2380483.324 | 10891260.853 |
|  |  | 95% Confidence Interval | Lower | 7966816.717 | 2094911.280 | 10303263.350 |
|  |  |  | Upper | 9054738.341 | 2666055.369 | 11479258.357 |
|  |  | Coefficient of Variation | | 0.033 | 0.061 | 0.027 |
|  |  | Unweighted Count | | 3839 | 1238 | 5077 |
|  | % within [Final] Known Hypertension | Estimate | | 78.1% | 21.9% | 100.0% |
|  |  | 95% Confidence Interval | Lower | 75.6% | 19.5% | 100.0% |
|  |  |  | Upper | 80.5% | 24.4% | 100.0% |
|  |  | Coefficient of Variation | | 0.016 | 0.056 | 0.000 |
|  |  | Unweighted Count | | 3839 | 1238 | 5077 |
| Yes | Population Size | Estimate | | 1113284.770 | 426347.586 | 1539632.356 |
|  |  | 95% Confidence Interval | Lower | 969907.105 | 358524.838 | 1366151.055 |
|  |  |  | Upper | 1256662.434 | 494170.335 | 1713113.658 |
|  |  | Coefficient of Variation | | 0.066 | 0.081 | 0.057 |
|  |  | Unweighted Count | | 767 | 337 | 1104 |
|  | % within [Final] Known Hypertension | Estimate | | 72.3% | 27.7% | 100.0% |
|  |  | 95% Confidence Interval | Lower | 68.6% | 24.3% | 100.0% |
|  |  |  | Upper | 75.7% | 31.4% | 100.0% |
|  |  | Coefficient of Variation | | 0.025 | 0.065 | 0.000 |
|  |  | Unweighted Count | | 767 | 337 | 1104 |
| Total | Population Size | Estimate | | 9624062.299 | 2806830.911 | 12430893.210 |
|  |  | 95% Confidence Interval | Lower | 9048895.497 | 2503200.626 | 11811329.257 |
|  |  |  | Upper | 10199229.100 | 3110461.195 | 13050457.162 |
|  |  | Coefficient of Variation | | 0.030 | 0.055 | 0.025 |
|  |  | Unweighted Count | | 4606 | 1575 | 6181 |
|  | % within [Final] Known Hypertension | Estimate | | 77.4% | 22.6% | 100.0% |
|  |  | 95% Confidence Interval | Lower | 75.1% | 20.4% | 100.0% |
|  |  |  | Upper | 79.6% | 24.9% | 100.0% |
|  |  | Coefficient of Variation | | 0.015 | 0.051 | 0.000 |
|  |  | Unweighted Count | | 4606 | 1575 | 6181 |

**S2 Table 11: Prevalence of impaired fasting glucose (IFG) by socio-demographic characteristics (Hypercholesterolemia)**

| **[Final] Known Hypercholesterolemia * CPG_impaired** | | | | | | |
| --- | --- | --- | --- | --- | --- | --- |
| [Final] Known Hypercholesterolemia | | | | CPG_impaired | | |
|  |  |  |  | no IFG | IFG 6.1-6.9 | Total |
| No | Population Size | Estimate | | 8705506.337 | 2423655.625 | 11129161.962 |
|  |  | 95% Confidence Interval | Lower | 8154244.474 | 2134905.660 | 10532793.025 |
|  |  |  | Upper | 9256768.199 | 2712405.590 | 11725530.898 |
|  |  | Coefficient of Variation | | 0.032 | 0.061 | 0.027 |
|  |  | Unweighted Count | | 3959 | 1276 | 5235 |
|  | % within [Final] Known Hypercholesterolemia | Estimate | | 78.2% | 21.8% | 100.0% |
|  |  | 95% Confidence Interval | Lower | 75.7% | 19.5% | 100.0% |
|  |  |  | Upper | 80.5% | 24.3% | 100.0% |
|  |  | Coefficient of Variation | | 0.016 | 0.056 | 0.000 |
|  |  | Unweighted Count | | 3959 | 1276 | 5235 |
| Yes | Population Size | Estimate | | 928114.237 | 383175.286 | 1311289.522 |
|  |  | 95% Confidence Interval | Lower | 817414.716 | 316453.443 | 1177918.752 |
|  |  |  | Upper | 1038813.758 | 449897.128 | 1444660.292 |
|  |  | Coefficient of Variation | | 0.061 | 0.089 | 0.052 |
|  |  | Unweighted Count | | 649 | 299 | 948 |
|  | % within [Final] Known Hypercholesterolemia | Estimate | | 70.8% | 29.2% | 100.0% |
|  |  | 95% Confidence Interval | Lower | 66.4% | 25.2% | 100.0% |
|  |  |  | Upper | 74.8% | 33.6% | 100.0% |
|  |  | Coefficient of Variation | | 0.030 | 0.073 | 0.000 |
|  |  | Unweighted Count | | 649 | 299 | 948 |
| Total | Population Size | Estimate | | 9633620.573 | 2806830.911 | 12440451.484 |
|  |  | 95% Confidence Interval | Lower | 9057507.820 | 2503200.626 | 11820016.270 |
|  |  |  | Upper | 10209733.326 | 3110461.195 | 13060886.698 |
|  |  | Coefficient of Variation | | 0.030 | 0.055 | 0.025 |
|  |  | Unweighted Count | | 4608 | 1575 | 6183 |
|  | % within [Final] Known Hypercholesterolemia | Estimate | | 77.4% | 22.6% | 100.0% |
|  |  | 95% Confidence Interval | Lower | 75.1% | 20.4% | 100.0% |
|  |  |  | Upper | 79.6% | 24.9% | 100.0% |
|  |  | Coefficient of Variation | | 0.015 | 0.051 | 0.000 |
|  |  | Unweighted Count | | 4608 | 1575 | 6183 |

**S2 Table 12: Prevalence of impaired fasting glucose (IFG) by socio-demographic characteristics (Physical Activity Level)**

| **[Final] Physical Activity Level (2 groups) * CPG_impaired** | | | | | | |
| --- | --- | --- | --- | --- | --- | --- |
| [Final] Physical Activity Level (2 groups) | | | | CPG_impaired | | |
|  |  |  |  | no IFG | IFG 6.1-6.9 | Total |
| Inactive | Population Size | Estimate | | 2278457.624 | 633984.440 | 2912442.064 |
|  |  | 95% Confidence Interval | Lower | 2032869.698 | 507056.919 | 2637549.972 |
|  |  |  | Upper | 2524045.551 | 760911.960 | 3187334.156 |
|  |  | Coefficient of Variation | | 0.055 | 0.102 | 0.048 |
|  |  | Unweighted Count | | 1081 | 374 | 1455 |
|  | % within [Final] Physical Activity Level (2 groups) | Estimate | | 78.2% | 21.8% | 100.0% |
|  |  | 95% Confidence Interval | Lower | 74.1% | 18.1% | 100.0% |
|  |  |  | Upper | 81.9% | 25.9% | 100.0% |
|  |  | Coefficient of Variation | | 0.025 | 0.091 | 0.000 |
|  |  | Unweighted Count | | 1081 | 374 | 1455 |
| Active | Population Size | Estimate | | 7251011.679 | 2146543.519 | 9397555.198 |
|  |  | 95% Confidence Interval | Lower | 6766456.538 | 1905846.784 | 8881459.945 |
|  |  |  | Upper | 7735566.819 | 2387240.254 | 9913650.451 |
|  |  | Coefficient of Variation | | 0.034 | 0.057 | 0.028 |
|  |  | Unweighted Count | | 3480 | 1190 | 4670 |
|  | % within [Final] Physical Activity Level (2 groups) | Estimate | | 77.2% | 22.8% | 100.0% |
|  |  | 95% Confidence Interval | Lower | 74.7% | 20.5% | 100.0% |
|  |  |  | Upper | 79.5% | 25.3% | 100.0% |
|  |  | Coefficient of Variation | | 0.016 | 0.054 | 0.000 |
|  |  | Unweighted Count | | 3480 | 1190 | 4670 |
| Total | Population Size | Estimate | | 9529469.303 | 2780527.959 | 12309997.262 |
|  |  | 95% Confidence Interval | Lower | 8954821.058 | 2477114.177 | 11690213.453 |
|  |  |  | Upper | 10104117.549 | 3083941.741 | 12929781.071 |
|  |  | Coefficient of Variation | | 0.031 | 0.056 | 0.026 |
|  |  | Unweighted Count | | 4561 | 1564 | 6125 |
|  | % within [Final] Physical Activity Level (2 groups) | Estimate | | 77.4% | 22.6% | 100.0% |
|  |  | 95% Confidence Interval | Lower | 75.1% | 20.4% | 100.0% |
|  |  |  | Upper | 79.6% | 24.9% | 100.0% |
|  |  | Coefficient of Variation | | 0.015 | 0.051 | 0.000 |
|  |  | Unweighted Count | | 4561 | 1564 | 6125 |

**S2 Table 13: Prevalence of impaired fasting glucose (IFG) by socio-demographic characteristics (Current Smokers)**

| **[Final] Current smokers * CPG_impaired** | | | | | | |
| --- | --- | --- | --- | --- | --- | --- |
| [Final] Current smokers | | | | CPG_impaired | | |
|  |  |  |  | no IFG | IFG 6.1-6.9 | Total |
| No | Population Size | Estimate | | 7281735.845 | 2180291.325 | 9462027.170 |
|  |  | 95% Confidence Interval | Lower | 6806979.280 | 1939219.911 | 8933593.131 |
|  |  |  | Upper | 7756492.410 | 2421362.739 | 9990461.210 |
|  |  | Coefficient of Variation | | 0.033 | 0.056 | 0.028 |
|  |  | Unweighted Count | | 3628 | 1277 | 4905 |
|  | % within [Final] Current smokers | Estimate | | 77.0% | 23.0% | 100.0% |
|  |  | 95% Confidence Interval | Lower | 74.6% | 20.8% | 100.0% |
|  |  |  | Upper | 79.2% | 25.4% | 100.0% |
|  |  | Coefficient of Variation | | 0.015 | 0.051 | 0.000 |
|  |  | Unweighted Count | | 3628 | 1277 | 4905 |
| Yes | Population Size | Estimate | | 2319157.598 | 614907.475 | 2934065.073 |
|  |  | 95% Confidence Interval | Lower | 2050301.662 | 482889.090 | 2640790.221 |
|  |  |  | Upper | 2588013.534 | 746925.861 | 3227339.926 |
|  |  | Coefficient of Variation | | 0.059 | 0.109 | 0.051 |
|  |  | Unweighted Count | | 965 | 295 | 1260 |
|  | % within [Final] Current smokers | Estimate | | 79.0% | 21.0% | 100.0% |
|  |  | 95% Confidence Interval | Lower | 74.6% | 17.1% | 100.0% |
|  |  |  | Upper | 82.9% | 25.4% | 100.0% |
|  |  | Coefficient of Variation | | 0.027 | 0.100 | 0.000 |
|  |  | Unweighted Count | | 965 | 295 | 1260 |
| Total | Population Size | Estimate | | 9600893.443 | 2795198.801 | 12396092.244 |
|  |  | 95% Confidence Interval | Lower | 9026262.687 | 2491886.860 | 11777446.282 |
|  |  |  | Upper | 10175524.199 | 3098510.741 | 13014738.206 |
|  |  | Coefficient of Variation | | 0.030 | 0.055 | 0.025 |
|  |  | Unweighted Count | | 4593 | 1572 | 6165 |
|  | % within [Final] Current smokers | Estimate | | 77.5% | 22.5% | 100.0% |
|  |  | 95% Confidence Interval | Lower | 75.1% | 20.4% | 100.0% |
|  |  |  | Upper | 79.6% | 24.9% | 100.0% |
|  |  | Coefficient of Variation | | 0.015 | 0.051 | 0.000 |
|  |  | Unweighted Count | | 4593 | 1572 | 6165 |

**S2 Table 14: Prevalence of impaired fasting glucose (IFG) by socio-demographic characteristics (P value)**

| **Tests of Independence (for P value)** | | | | | | |
| --- | --- | --- | --- | --- | --- | --- |
|  | | Chi-Square | Adjusted F | df1 | df2 | Sig. |
| [Final] Locality * CPG_impaired | Pearson | 2.261 | 0.668 | 1 | 442 | 0.414 |
|  | Likelihood Ratio | 2.283 | 0.675 | 1 | 442 | 0.412 |
| [Final] Gender * CPG_impaired | Pearson | 3.173 | 1.229 | 1 | 442 | 0.268 |
|  | Likelihood Ratio | 3.172 | 1.228 | 1 | 442 | 0.268 |
| Age Recode 3 group * CPG_impaired | Pearson | 57.265 | 14.787 | 1.937 | 856.309 | 0.000 |
|  | Likelihood Ratio | 55.855 | 14.423 | 1.937 | 856.309 | 0.000 |
| Race Recode 4 grp * CPG_impaired | Pearson | 43.009 | 3.576 | 2.822 | 1247.317 | 0.015 |
|  | Likelihood Ratio | 44.899 | 3.733 | 2.822 | 1247.317 | 0.013 |
| Marital Status 2group * CPG_impaired | Pearson | 51.756 | 20.122 | 1 | 442 | 0.000 |
|  | Likelihood Ratio | 52.853 | 20.549 | 1 | 442 | 0.000 |
| Education Recode 3 Group * CPG_impaired | Pearson | 15.453 | 2.659 | 1.983 | 876.350 | 0.071 |
|  | Likelihood Ratio | 15.638 | 2.691 | 1.983 | 876.350 | 0.069 |
| [Final] Household Income - By state cut off, DOSM 2016 * CPG_impaired | Pearson | 8.484 | 1.542 | 1.992 | 880.383 | 0.215 |
|  | Likelihood Ratio | 8.680 | 1.577 | 1.992 | 880.383 | 0.207 |
| [Final] Known Hypertension * CPG_impaired | Pearson | 13.062 | 9.558 | 1 | 442 | 0.002 |
|  | Likelihood Ratio | 12.532 | 9.170 | 1 | 442 | 0.003 |
| [Final] Known Hypercholesterolemia * CPG_impaired | Pearson | 18.490 | 11.741 | 1 | 442 | 0.001 |
|  | Likelihood Ratio | 17.516 | 11.123 | 1 | 442 | 0.001 |
| [Final] Current smokers * CPG_impaired | Pearson | 2.773 | 0.987 | 1 | 442 | 0.321 |
|  | Likelihood Ratio | 2.806 | 0.999 | 1 | 442 | 0.318 |
| BMI WHO 3 group * CPG_impaired | Pearson | 37.238 | 7.812 | 1.963 | 867.832 | 0.000 |
|  | Likelihood Ratio | 37.247 | 7.814 | 1.963 | 867.832 | 0.000 |
| [Final] Physical Activity Level (2 groups) * CPG_impaired | Pearson | 0.729 | 0.278 | 1 | 442 | 0.598 |
|  | Likelihood Ratio | 0.733 | 0.280 | 1 | 442 | 0.597 |
| The adjusted F is a variant of the second-order Rao-Scott adjusted chi-square statistic. Significance is based on the adjusted F and its degrees of freedom. | | | | | | |

**S3 Table 1: Factors associated with Impaired Fasting Glycemia Among the Malaysian Adult Population (Locality: OR(95%CI)**

- All tables in S3 correspond to Table 3 in the manuscript

| **Parameter Estimates** | | | | | | | | | | |
| --- | --- | --- | --- | --- | --- | --- | --- | --- | --- | --- |
| impaired FBS (6.1-6.9) dan no diabetic | Parameter | B | 95% Confidence Interval | | Hypothesis Test | | | Exp(B) | 95% Confidence Interval for Exp(B) | |
|  |  |  | Lower | Upper | t | df | Sig. |  | Lower | Upper |
| IFG 6.1-6.9 | (Intercept) | -1.314 | -1.517 | -1.111 | -12.700 | 442.000 | 0.000 | 0.269 | 0.219 | 0.329 |
|  | [strata_gp=1] | 0.107 | -0.150 | 0.364 | 0.817 | 442.000 | 0.414 | 1.113 | 0.860 | 1.439 |
|  | [strata_gp=2] | .000^a^ |  |  |  |  |  | 1.000 |  |  |

Dependent Variable: impaired FBS (6.1-6.9) dan no diabetic (reference category = no IFG)
Model: (Intercept), strata_gp

**S3 Table 2: Factors associated with Impaired Fasting Glycemia Among the Malaysian Adult Population (Sex: OR(95%CI)**

| **Parameter Estimates** | | | | | | | | | | |
| --- | --- | --- | --- | --- | --- | --- | --- | --- | --- | --- |
| impaired FBS (6.1-6.9) dan no diabetic | Parameter | B | 95% Confidence Interval | | Hypothesis Test | | | Exp(B) | 95% Confidence Interval for Exp(B) | |
|  |  |  | Lower | Upper | t | df | Sig. |  | Lower | Upper |
| IFG 6.1-6.9 | (Intercept) | -1.287 | -1.451 | -1.122 | -15.403 | 442.000 | 0.000 | 0.276 | 0.234 | 0.325 |
|  | [logit_gender=1.00] | 0.108 | -0.084 | 0.301 | 1.108 | 442.000 | 0.268 | 1.114 | 0.920 | 1.351 |
|  | [logit_gender=2.00] | .000^a^ |  |  |  |  |  | 1.000 |  |  |
| Dependent Variable: impaired FBS (6.1-6.9) dan no diabetic (reference category = no IFG)  Model: (Intercept), logit_gender | | | | | | | | | | |

**S3 Table 3: Factors associated with Impaired Fasting Glycemia Among the Malaysian Adult Population (Age group: OR(95%CI)**

| **Parameter Estimates** | | | | | | | | | | |
| --- | --- | --- | --- | --- | --- | --- | --- | --- | --- | --- |
| impaired FBS (6.1-6.9) dan no diabetic | Parameter | B | 95% Confidence Interval | | Hypothesis Test | | | Exp(B) | 95% Confidence Interval for Exp(B) | |
|  |  |  | Lower | Upper | t | df | Sig. |  | Lower | Upper |
| IFG 6.1-6.9 | (Intercept) | -1.417 | -1.592 | -1.243 | -15.964 | 442.000 | 0.000 | 0.242 | 0.204 | 0.289 |
|  | [logit_age=1.00] | 0.583 | 0.346 | 0.819 | 4.839 | 442.000 | 0.000 | 1.791 | 1.413 | 2.269 |
|  | [logit_age=2.00] | 0.376 | 0.173 | 0.580 | 3.639 | 442.000 | 0.000 | 1.457 | 1.189 | 1.785 |
|  | [logit_age=3.00] | .000^a^ |  |  |  |  |  | 1.000 |  |  |
| Dependent Variable: impaired FBS (6.1-6.9) dan no diabetic (reference category = no IFG)  Model: (Intercept), logit_age | | | | | | | | | | |

**S3 Table 4: Factors associated with Impaired Fasting Glycemia Among the Malaysian Adult Population (Ethnicity: OR(95%CI)**

| **Parameter Estimates** | | | | | | | | | | |
| --- | --- | --- | --- | --- | --- | --- | --- | --- | --- | --- |
| impaired FBS (6.1-6.9) dan no diabetic | Parameter | B | 95% Confidence Interval | | Hypothesis Test | | | Exp(B) | 95% Confidence Interval for Exp(B) | |
|  |  |  | Lower | Upper | t | df | Sig. |  | Lower | Upper |
| IFG 6.1-6.9 | (Intercept) | -1.233 | -1.731 | -0.734 | -4.862 | 442.000 | 0.000 | 0.292 | 0.177 | 0.480 |
|  | [logit_race=1.00] | 0.101 | -0.418 | 0.619 | 0.382 | 442.000 | 0.702 | 1.106 | 0.659 | 1.858 |
|  | [logit_race=2.00] | 0.167 | -0.409 | 0.742 | 0.570 | 442.000 | 0.569 | 1.182 | 0.665 | 2.101 |
|  | [logit_race=3.00] | -0.369 | -0.928 | 0.190 | -1.297 | 442.000 | 0.195 | 0.691 | 0.395 | 1.210 |
|  | [logit_race=4.00] | .000^a^ |  |  |  |  |  | 1.000 |  |  |
| Dependent Variable: impaired FBS (6.1-6.9) dan no diabetic (reference category = no IFG)  Model: (Intercept), logit_race | | | | | | | | | | |

**S3 Table 5: Factors associated with Impaired Fasting Glycemia Among the Malaysian Adult Population (Marital Status: OR(95%CI)**

| **Parameter Estimates** | | | | | | | | | | |
| --- | --- | --- | --- | --- | --- | --- | --- | --- | --- | --- |
| impaired FBS (6.1-6.9) dan no diabetic | Parameter | B | 95% Confidence Interval | | Hypothesis Test | | | Exp(B) | 95% Confidence Interval for Exp(B) | |
|  |  |  | Lower | Upper | t | df | Sig. |  | Lower | Upper |
| IFG 6.1-6.9 | (Intercept) | -1.525 | -1.704 | -1.345 | -16.677 | 442.000 | 0.000 | 0.218 | 0.182 | 0.261 |
|  | [logit_marital=1.00] | 0.462 | 0.259 | 0.666 | 4.465 | 442.000 | 0.000 | 1.588 | 1.295 | 1.946 |
|  | [logit_marital=2.00] | .000^a^ |  |  |  |  |  | 1.000 |  |  |
| Dependent Variable: impaired FBS (6.1-6.9) dan no diabetic (reference category = no IFG)  Model: (Intercept), logit_marital | | | | | | | | | | |

**S3 Table 6: Factors associated with Impaired Fasting Glycemia Among the Malaysian Adult Population (Educational Status: OR(95%CI)**

| **Parameter Estimates** | | | | | | | | | | |
| --- | --- | --- | --- | --- | --- | --- | --- | --- | --- | --- |
| impaired FBS (6.1-6.9) dan no diabetic | Parameter | B | 95% Confidence Interval | | Hypothesis Test | | | Exp(B) | 95% Confidence Interval for Exp(B) | |
|  |  |  | Lower | Upper | t | df | Sig. |  | Lower | Upper |
| IFG 6.1-6.9 | (Intercept) | -1.418 | -1.616 | -1.220 | -14.047 | 442.000 | 0.000 | 0.242 | 0.199 | 0.295 |
|  | [logit_edu=1.00] | 0.292 | 0.051 | 0.533 | 2.383 | 442.000 | 0.018 | 1.339 | 1.053 | 1.703 |
|  | [logit_edu=2.00] | 0.154 | -0.144 | 0.452 | 1.015 | 442.000 | 0.311 | 1.166 | 0.866 | 1.571 |
|  | [logit_edu=3.00] | .000^a^ |  |  |  |  |  | 1.000 |  |  |
| Dependent Variable: impaired FBS (6.1-6.9) dan no diabetic (reference category = no IFG)  Model: (Intercept), logit_edu | | | | | | | | | | |

**S3 Table 7: Factors associated with Impaired Fasting Glycemia Among the Malaysian Adult Population (Household Income: OR(95%CI)**

| **Parameter Estimates** | | | | | | | | | | |
| --- | --- | --- | --- | --- | --- | --- | --- | --- | --- | --- |
| impaired FBS (6.1-6.9) dan no diabetic | Parameter | B | 95% Confidence Interval | | Hypothesis Test | | | Exp(B) | 95% Confidence Interval for Exp(B) | |
|  |  |  | Lower | Upper | t | df | Sig. |  | Lower | Upper |
| IFG 6.1-6.9 | (Intercept) | -1.493 | -1.848 | -1.139 | -8.286 | 442.000 | 0.000 | 0.225 | 0.158 | 0.320 |
|  | [Household=1] | 0.293 | -0.091 | 0.677 | 1.502 | 442.000 | 0.134 | 1.341 | 0.913 | 1.968 |
|  | [Household=2] | 0.157 | -0.246 | 0.560 | 0.766 | 442.000 | 0.444 | 1.170 | 0.782 | 1.751 |
|  | [Household=3] | .000^a^ |  |  |  |  |  | 1.000 |  |  |
| Dependent Variable: impaired FBS (6.1-6.9) dan no diabetic (reference category = no IFG)  Model: (Intercept), Household | | | | | | | | | | |

**S3 Table 8: Factors associated with Impaired Fasting Glycemia Among the Malaysian Adult Population (BMI: OR(95%CI)**

| **Parameter Estimates** | | | | | | | | | | |
| --- | --- | --- | --- | --- | --- | --- | --- | --- | --- | --- |
| impaired FBS (6.1-6.9) dan no diabetic | Parameter | B | 95% Confidence Interval | | Hypothesis Test | | | Exp(B) | 95% Confidence Interval for Exp(B) | |
|  |  |  | Lower | Upper | t | df | Sig. |  | Lower | Upper |
| IFG 6.1-6.9 | (Intercept) | -1.515 | -1.910 | -1.121 | -7.554 | 441.000 | 0.000 | 0.220 | 0.148 | 0.326 |
|  | [logit_BMI=1.00] | 0.456 | 0.051 | 0.861 | 2.214 | 441.000 | 0.027 | 1.578 | 1.053 | 2.366 |
|  | [logit_BMI=2.00] | 0.092 | -0.292 | 0.477 | 0.472 | 441.000 | 0.637 | 1.097 | 0.747 | 1.611 |
|  | [logit_BMI=3.00] | .000^a^ |  |  |  |  |  | 1.000 |  |  |
| Dependent Variable: impaired FBS (6.1-6.9) dan no diabetic (reference category = no IFG)  Model: (Intercept), logit_BMI | | | | | | | | | | |

**S3 Table 9: Factors associated with Impaired Fasting Glycemia Among the Malaysian Adult Population (Hypertension: OR(95%CI)**

| **Parameter Estimates** | | | | | | | | | | |
| --- | --- | --- | --- | --- | --- | --- | --- | --- | --- | --- |
| impaired FBS (6.1-6.9) dan no diabetic | Parameter | B | 95% Confidence Interval | | Hypothesis Test | | | Exp(B) | 95% Confidence Interval for Exp(B) | |
|  |  |  | Lower | Upper | t | df | Sig. |  | Lower | Upper |
| IFG 6.1-6.9 | (Intercept) | -1.274 | -1.416 | -1.132 | -17.687 | 442.000 | 0.000 | 0.280 | 0.243 | 0.322 |
|  | [logitHPT=1.00] | 0.314 | 0.114 | 0.515 | 3.082 | 442.000 | 0.002 | 1.369 | 1.121 | 1.673 |
|  | [logitHPT=2.00] | .000^a^ |  |  |  |  |  | 1.000 |  |  |
| Dependent Variable: impaired FBS (6.1-6.9) dan no diabetic (reference category = no IFG)  Model: (Intercept), logitHPT | | | | | | | | | | |

**S3 Table 10: Factors associated with Impaired Fasting Glycemia Among the Malaysian Adult Population (Hypercholesterolemia: OR(95%CI)**

| **Parameter Estimates** | | | | | | | | | | |
| --- | --- | --- | --- | --- | --- | --- | --- | --- | --- | --- |
| impaired FBS (6.1-6.9) dan no diabetic | Parameter | B | 95% Confidence Interval | | Hypothesis Test | | | Exp(B) | 95% Confidence Interval for Exp(B) | |
|  |  |  | Lower | Upper | t | df | Sig. |  | Lower | Upper |
| IFG 6.1-6.9 | (Intercept) | -1.279 | -1.419 | -1.138 | -17.904 | 442.000 | 0.000 | 0.278 | 0.242 | 0.320 |
|  | [logitHPL=1.00] | 0.394 | 0.167 | 0.621 | 3.410 | 442.000 | 0.001 | 1.483 | 1.182 | 1.861 |
|  | [logitHPL=2.00] | .000^a^ |  |  |  |  |  | 1.000 |  |  |
| Dependent Variable: impaired FBS (6.1-6.9) dan no diabetic (reference category = no IFG)  Model: (Intercept), logitHPL | | | | | | | | | | |

**S3 Table 11: Factors associated with Impaired Fasting Glycemia Among the Malaysian Adult Population (Physical Activity Level: OR(95%CI)**

| **Parameter Estimates** | | | | | | | | | | |
| --- | --- | --- | --- | --- | --- | --- | --- | --- | --- | --- |
| impaired FBS (6.1-6.9) dan no diabetic | Parameter | B | 95% Confidence Interval | | Hypothesis Test | | | Exp(B) | 95% Confidence Interval for Exp(B) | |
|  |  |  | Lower | Upper | t | df | Sig. |  | Lower | Upper |
| IFG 6.1-6.9 | (Intercept) | -1.279 | -1.508 | -1.051 | -10.994 | 442.000 | 0.000 | 0.278 | 0.221 | 0.350 |
|  | [logit_PA=1.00] | 0.062 | -0.169 | 0.293 | 0.527 | 442.000 | 0.598 | 1.064 | 0.845 | 1.340 |
|  | [logit_PA=2.00] | .000^a^ |  |  |  |  |  | 1.000 |  |  |
| Dependent Variable: impaired FBS (6.1-6.9) dan no diabetic (reference category = no IFG)  Model: (Intercept), logit_PA | | | | | | | | | | |

**S3 Table 12: Factors associated with Impaired Fasting Glycemia Among the Malaysian Adult Population (Current Smokers: OR(95%CI)**

| **Parameter Estimates** | | | | | | | | | | |
| --- | --- | --- | --- | --- | --- | --- | --- | --- | --- | --- |
| impaired FBS (6.1-6.9) dan no diabetic | Parameter | B | 95% Confidence Interval | | Hypothesis Test | | | Exp(B) | 95% Confidence Interval for Exp(B) | |
|  |  |  | Lower | Upper | t | df | Sig. |  | Lower | Upper |
| IFG 6.1-6.9 | (Intercept) | -1.206 | -1.335 | -1.077 | -18.315 | 442.000 | 0.000 | 0.299 | 0.263 | 0.341 |
|  | [logit_smoker=1.00] | -0.122 | -0.362 | 0.119 | -0.993 | 442.000 | 0.321 | 0.886 | 0.696 | 1.126 |
|  | [logit_smoker=2.00] | .000^a^ |  |  |  |  |  | 1.000 |  |  |
| Dependent Variable: impaired FBS (6.1-6.9) dan no diabetic (reference category = no IFG)  Model: (Intercept), logit_smoker | | | | | | | | | | |

**S3 Table 13: Factors associated with Impaired Fasting Glycemia Among the Malaysian Adult Population (aOR(95%CI)**

| **Parameter Estimates (Table 3: aOR)** | | | | | | | | | | |
| --- | --- | --- | --- | --- | --- | --- | --- | --- | --- | --- |
| impaired FBS (6.1-6.9) dan no diabetic | Parameter | B | 95% Confidence Interval | | Hypothesis Test | | | Exp(B) | 95% Confidence Interval for Exp(B) | |
|  |  |  | Lower | Upper | t | df | Sig. |  | Lower | Upper |
| IFG 6.1-6.9 | (Intercept) | -2.088 | -2.872 | -1.305 | -5.239 | 441.000 | 0.000 | 0.124 | 0.057 | 0.271 |
|  | [strata_gp=1] | 0.056 | -0.224 | 0.336 | 0.396 | 441.000 | 0.692 | 1.058 | 0.800 | 1.400 |
|  | [strata_gp=2] | .000^a^ |  |  |  |  |  | 1.000 |  |  |
|  | [logit_gender=1.00] | 0.006 | -0.221 | 0.233 | 0.051 | 441.000 | 0.960 | 1.006 | 0.802 | 1.262 |
|  | [logit_gender=2.00] | .000^a^ |  |  |  |  |  | 1.000 |  |  |
|  | [logit_age=1.00] | 0.409 | 0.094 | 0.724 | 2.550 | 441.000 | 0.011 | 1.505 | 1.098 | 2.062 |
|  | [logit_age=2.00] | 0.157 | -0.070 | 0.385 | 1.358 | 441.000 | 0.175 | 1.170 | 0.932 | 1.470 |
|  | [logit_age=3.00] | .000^a^ |  |  |  |  |  | 1.000 |  |  |
|  | [logit_race=1.00] | 0.123 | -0.418 | 0.663 | 0.446 | 441.000 | 0.656 | 1.130 | 0.658 | 1.941 |
|  | [logit_race=2.00] | -0.004 | -0.601 | 0.593 | -0.014 | 441.000 | 0.989 | 0.996 | 0.548 | 1.809 |
|  | [logit_race=3.00] | -0.409 | -0.977 | 0.160 | -1.413 | 441.000 | 0.158 | 0.665 | 0.376 | 1.173 |
|  | [logit_race=4.00] | .000^a^ |  |  |  |  |  | 1.000 |  |  |
|  | [logit_marital=1.00] | 0.377 | 0.148 | 0.607 | 3.235 | 441.000 | 0.001 | 1.458 | 1.160 | 1.834 |
|  | [logit_marital=2.00] | .000^a^ |  |  |  |  |  | 1.000 |  |  |
|  | [logit_edu=1.00] | 0.142 | -0.115 | 0.399 | 1.083 | 441.000 | 0.279 | 1.152 | 0.891 | 1.490 |
|  | [logit_edu=2.00] | 0.039 | -0.318 | 0.397 | 0.217 | 441.000 | 0.828 | 1.040 | 0.728 | 1.487 |
|  | [logit_edu=3.00] | .000^a^ |  |  |  |  |  | 1.000 |  |  |
|  | [Household=1] | 0.296 | -0.122 | 0.714 | 1.392 | 441.000 | 0.165 | 1.344 | 0.885 | 2.041 |
|  | [Household=2] | 0.147 | -0.291 | 0.585 | 0.659 | 441.000 | 0.510 | 1.158 | 0.748 | 1.794 |
|  | [Household=3] | .000^a^ |  |  |  |  |  | 1.000 |  |  |
|  | [logitHPT=1.00] | -0.112 | -0.378 | 0.155 | -0.822 | 441.000 | 0.411 | 0.894 | 0.685 | 1.168 |
|  | [logitHPT=2.00] | .000^a^ |  |  |  |  |  | 1.000 |  |  |
|  | [logitHPL=1.00] | 0.271 | -0.035 | 0.576 | 1.742 | 441.000 | 0.082 | 1.311 | 0.966 | 1.780 |
|  | [logitHPL=2.00] | .000^a^ |  |  |  |  |  | 1.000 |  |  |
|  | [logit_smoker=1.00] | -0.045 | -0.320 | 0.230 | -0.322 | 441.000 | 0.748 | 0.956 | 0.726 | 1.259 |
|  | [logit_smoker=2.00] | .000^a^ |  |  |  |  |  | 1.000 |  |  |
|  | [logit_BMI=1.00] | 0.215 | -0.206 | 0.636 | 1.003 | 441.000 | 0.316 | 1.240 | 0.814 | 1.889 |
|  | [logit_BMI=2.00] | -0.119 | -0.520 | 0.283 | -0.580 | 441.000 | 0.562 | 0.888 | 0.595 | 1.327 |
|  | [logit_BMI=3.00] | .000^a^ |  |  |  |  |  | 1.000 |  |  |
|  | [logit_PA=1.00] | 0.125 | -0.132 | 0.382 | 0.956 | 441.000 | 0.340 | 1.133 | 0.876 | 1.465 |
|  | [logit_PA=2.00] | .000^a^ |  |  |  |  |  | 1.000 |  |  |
| Dependent Variable: impaired FBS (6.1-6.9) dan no diabetic (reference category = no IFG)  Model: (Intercept), strata_gp, logit_gender, logit_age, logit_race, logit_marital, logit_edu, Household, logitHPT, logitHPL, logit_smoker, logit_BMI, logit_PA | | | | | | | | | | |
